# Circulating biomarkers of cardiovascular disease are related to aneurysm volume in abdominal aortic aneurysm

**DOI:** 10.1177/1358863X231181159

**Published:** 2023-07-03

**Authors:** Elke Bouwens, Alexander Vanmaele, Sanne E Hoeks, Hence JM Verhagen, Bram Fioole, Adriaan Moelker, Sander ten Raa, Burhan Hussain, José Oliveira-Pinto, Frederico Bastos Gonçalves, Arne S Ijpma, Imo E Hoefer, Felix van Lier, K Martijn Akkerhuis, Danielle F Majoor-Krakauer, Eric Boersma, Isabella Kardys

**Affiliations:** 1 Department of Cardiology, Erasmus MC, Rotterdam, The Netherlands; 2 Department of Vascular Surgery, Erasmus MC, Rotterdam, The Netherlands; 3 Department of Anesthesiology, Erasmus MC, Rotterdam, The Netherlands; 4 Department of Vascular Surgery, Maasstad Hospital, Rotterdam, The Netherlands; 5 Department of Radiology and Nuclear Medicine, Erasmus MC, Rotterdam, The Netherlands; 6 Department of Radiology, Beatrix Hospital, Gorinchem, The Netherlands; 7 Department of Angiology and Vascular Surgery, Centro Hospitalar São João, Porto, Portugal; 8 Department of Surgery and Physiology, Faculty of Medicine of Oporto, Porto, Portugal; 9 NOVA Medical School, Universidade NOVA de Lisboa, Lisbon, Portugal; 10 Department of Angiology and Vascular Surgery, Hospital de Santa Marta, Centro Hospitalar Universitário de Lisboa Central, Lisbon, Portugal; 11 Department of Clinical Genetics, Erasmus MC, Rotterdam, The Netherlands; 12 Central Diagnostic Laboratory, University Medical Center Utrecht, Utrecht, The Netherlands

**Keywords:** abdominal aortic aneurysm (AAA), biomarkers, cardiovascular diseases, endovascular aneurysm repair (EVAR)

## Abstract

**Background:**

Surveillance programs in abdominal aortic aneurysms (AAA) are mainly based on imaging and leave room for improvement to timely identify patients at risk for AAA growth. Many biomarkers are dysregulated in patients with AAA, which fuels interest in biomarkers as indicators of disease progression. We examined associations of 92 cardiovascular disease (CVD)-related circulating biomarkers with AAA and sac volume.

**Methods:**

In a cross-sectional analysis, we separately investigated (1) 110 watchful waiting (WW) patients (undergoing periodic surveillance imaging without planned intervention) and (2) 203 patients after endovascular aneurysm repair (EVAR). The Cardiovascular Panel III (Olink Proteomics AB, Sweden) was used to measure 92 CVD-related circulating biomarkers. We used cluster analyses to investigate protein-based subphenotypes, and linear regression to examine associations of biomarkers with AAA and sac volume on CT scans.

**Results:**

Cluster analyses revealed two biomarker-based subgroups in both WW and EVAR patients, with higher levels of 76 and 74 proteins, respectively, in one subgroup versus the other. In WW patients, uPA showed a borderline significant association with AAA volume. Adjusting for clinical characteristics, there was a difference of −0.092 (−0.148, −0.036) log_e_ mL in AAA volume per SD uPA. In EVAR patients, after multivariable adjustment, four biomarkers remained significantly associated with sac volume. The mean effects on sac volume per SD difference were: LDLR: −0.128 (−0.212, −0.044), TFPI: 0.139 (0.049, 0.229), TIMP4: 0.110 (0.023, 0.197), IGFBP-2: 0.103 (0.012, 0.194).

**Conclusion:**

LDLR, TFPI, TIMP4, and IGFBP-2 were independently associated with sac volume after EVAR. Subgroups of patients with high levels of the majority of CVD-related biomarkers emphasize the intertwined relationship between AAA and CVD.

**ClinicalTrials.gov Identifier: NCT03703947.**

## Background

The natural course of abdominal aortic aneurysms (AAAs) is gradual expansion over time, with rupture as the most disastrous and frequently fatal complication.^1^ Elective open or endovascular repair is considered for large or rapidly growing AAAs, in an attempt to prevent mortality due to ruptured AAA.^2^ However, due to individual differences in growth rate, only a distinct group of AAA patients will eventually need such surgical repair.^3^ This stresses the importance of improving the current, uniform surveillance policies into personalized protocols. Similarly, the strict surveillance protocol after endovascular aneurysm repair (EVAR) is known to be quite inefficient.^4^ Attempts have been made to individualize post-EVAR follow up, and sac dynamics has been identified as a key factor. Patients demonstrating early sac shrinkage after EVAR show a low adverse event risk and prolonged survival, although the biological mechanisms and variation in sac dynamics among patients remain unexplained.^4,5^

Circulating biomarkers may reflect disease activity, may improve diagnostic and prognostic precision, and may even lead to identification of pathophysiological processes or therapeutic options.^6,7^ Owing to ease of use, biomarker testing in cardiac diseases, such as coronary artery disease and heart failure, is incorporated into diagnosis and risk stratification.^8^ Common clinical markers, such as C-reactive protein (CRP) and D-dimer, have been most extensively investigated as potential biomarkers for AAA growth, but results have been inconsistent and these markers lack the desired specificity.^9^ In order to look beyond the likely candidates, a broader approach is required.^7^ Given the overlap in biological systems and risk factors involved, and the ensuing strong correlation between AAAs and coronary artery disease,^10^ circulating cardiovascular biomarkers hold promise for predicting disease progression in AAA as well. More exploratory AAA research with limited sets of markers has identified several biomarker candidates that reflect AAA progression, including metalloproteinases and their inhibitors, markers in anticoagulation, fibrinolysis, and lipid homeostasis.^7,9,11^ Aside the methodological heterogeneity and often limited sample sizes, these studies almost exclusively investigated either one or a limited number of preselected biomarkers.^9^

The aim of this study was to examine associations of a broad range of circulating biomarkers that have been linked previously to other cardiovascular diseases with AAA/sac volume. Associations were examined separately for patients under a ‘watchful waiting’ policy and for patients after EVAR, because these groups represent distinct stages of the disease process, with different biological mechanisms within the aneurysm sac. Furthermore, we performed cluster analysis to investigate whether there are subgroups of patients based on protein levels and whether these subgroups have particular AAA characteristics.

## Methods

### Study participants

**Figure 1.**
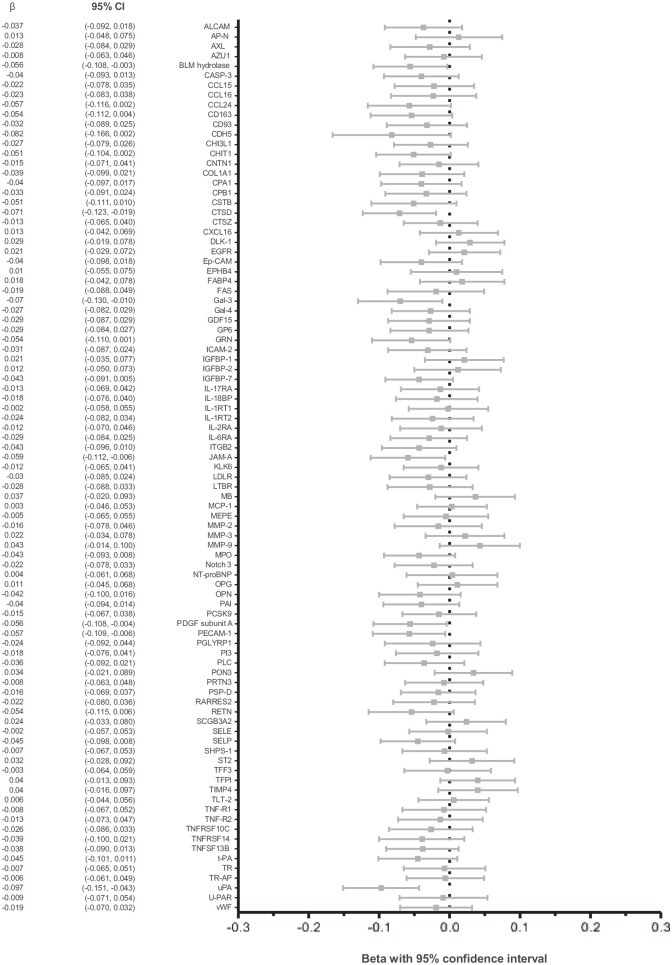
Association of biomarkers with aneurysm volume in watchful waiting patients. This figure represents the results of univariable linear regressions where we examined associations of individual biomarkers with AAA volume for the watchful waiting patients. Results are presented as the mean effect (β) with 95% CI of a 1 SD difference of the biomarker on AAA volume (expressed as natural log mL). AAA, abdominal aortic aneurysm.

The ‘Study of biomarker profiling to unravel the intertwined pathophysiology of coronary artery disease and abdominal aortic aneurysm’ (BIOMArCS-AAA) is an ongoing observational, multicenter study, which aims to investigate the longitudinal association between circulating biomarkers and AAA growth. Patients are recruited through the vascular surgery outpatient clinics of two hospitals in The Netherlands. Inclusion/exclusion criteria, sample size calculation, and the full study design with repeated blood sampling and imaging are depicted in the online supplementary material and Figure 1. Results of the longitudinal data, including association with AAA growth or sac shrinkage, and clinical endpoints, will follow in a later stage, after completion of follow up. In the current cross-sectional analysis, which is a pilot for the future longitudinal investigation, we focused on a single study visit of 340 patients enrolled between March 2017 and May 2020. These were (1) patients under a watchful waiting policy (periodic clinical observation, no need for aneurysm repair in the near future) and (2) patients who had undergone EVAR for AAA. Of the 340 available patients, 24 were excluded due to incomplete computed tomography (CT) imaging or blood sample data (supplemental Figure S1A). Hence, the current analysis consists of 316 patients for laboratory analysis (113 watchful waiting and 203 EVAR, which will be analyzed separately). BIOMArCS-AAA was approved by the medical ethics committee and conducted in accordance with the Declaration of Helsinki. Written informed consent was obtained from all patients.

### Study procedures

BIOMArCS-AAA is an observational study, and all patients were treated according to the discretion of the treating physician, based on prevailing guidelines. Clinical characteristics were collected at study baseline. We collected study material (blood sample and CT scan) at baseline for the watchful waiting patients, and for the EVAR patients immediately after endograft implantation (supplemental Figure S2).

### CT scanning

A CT report for standard medical care was provided by the radiologist, including maximal diameter. Additional aneurysm and sac measurements were obtained from the CT scan by semi-automatically generated center lumen line reconstructions performed on dedicated reconstruction software (3mensio Vascular; Medical Imaging BV, Bilthoven, The Netherlands) with previously demonstrated high inter and intra-observer agreement.^12^ When CT scans were performed in the context of standard medical care, these were used for study purposes. If not available, additional (noncontrast) CT scans were conducted.

### Blood sampling

Blood samples were stored at −80°C until analysis. Analysis was performed in one batch (Central Diagnostic Laboratory, University Medical Center Utrecht, The Netherlands) using the Cardiovascular III panel of Olink Proteomics AB (Uppsala, Sweden), which is a multiplex immunoassay, quantifying 92 protein biomarkers simultaneously using proximity extension assay technology.^13^ Biomarkers on the assay were chosen based on their potential to represent aspects of cardiovascular pathophysiology, including both known cardiovascular and inflammatory biomarkers as well as exploratory proteins with potential as new biomarkers. Protein abundance is measured on 1 µL EDTA plasma by a DNA polymerase chain reaction, based on the binding of two specific antibodies to their respective target proteins in the sample. Four internal controls were added to each assay to reduce intra-assay variability and, additionally, two external controls were also included on each plate to improve inter-assay precision (online supplementary text).^14^ Intra- and inter-assay coefficient of variation were 6% and 12%, respectively. Biomarkers are expressed in Normalized Protein eXpression (NPX), which are relative units that result from the PCR and which are on the log_2_ scale. A high NPX value represents a high biomarker concentration. We standardized NPX values as Z-scores. Biomarker Spondin-1 had a low expression in the majority of our blood samples (below limit of detection in 90%) and was therefore excluded from further analyses. The investigated biomarkers are described in supplemental Table S1.

### Outcome measures

The primary outcome for this investigation was total aneurysm or sac volume and this was measured according to a previously validated method comprising lumen, intraluminal thrombus, calcification, and aortic wall.^15^ It was measured from 10 mm distal to the lowermost renal artery to 10 mm above the aortic bifurcation, as described before.^16^ A subset of 27 randomly selected CT scans was reassessed to calculate the intraobserver variability, with a minimal interval of 12 days between both measurements. To calculate the interobserver variability, a randomly selected set of 41 CT scans were reassessed by an independent observer. The intraclass correlation coefficient was calculated to assess both variability measures (two-way mixed-effects model, absolute agreement).

### Statistical analysis

In the primary analyses, we examined patients under a watchful waiting policy and patients after EVAR separately because the ongoing biological processes differ between these groups. Thus, we focused on within-group associations. Continuous variables with a normal distribution are presented as mean ± SD, or median (25th to 75th percentile) in case of nonnormality. Categorical variables are presented as counts and percentages, and differences between groups were tested using the chi-squared test or Fisher’s exact test, as appropriate.

An extensive description of the statistical analyses can be found in the online supplementary material. In brief, we performed k-means clustering to examine the presence of subgroups based solely on the biomarker profile.^17^ We examined the distribution of clinical characteristics and aneurysm-related measurements according to the clusters, and differences were tested with Student’s *t*-tests or Mann–Whitney *U*-tests depending on distributions. Pathways underlying the differences in biomarker levels between clusters were investigated by Ingenuity Pathway Analysis, using a multiple testing adjusted *p*-value of 0.05 as the threshold for variable selection and comparing the expression of these selected proteins against the currently used biomarker panel. We used heatmaps to visualize the biomarker results for each cluster.

Subsequently, we examined associations of individual biomarkers with AAA/sac volume (again separately in watchful waiting patients and in patients after EVAR). Results are presented as the mean effect with 95% CI of a 1 SD difference of the biomarker level on AAA/sac volume (expressed as natural log (log_e_) mL). First, univariable linear regression analyses were performed. Subsequently, age, sex, and body surface area (BSA) were added to the model (A). For patients after EVAR surgery, time between EVAR surgery and study visit was also added to the model (B). Finally, additional predefined variables were added (C): history of coronary heart disease, hypertension, diabetes mellitus, smoking status (current or former smokers vs never smoked), peripheral artery occlusive disease, antiplatelet therapy, lipid-lowering drug therapy, and familial AAA (defined as at least one first-degree relative affected by aortic aneurysm, based on anamnestic information). Ultimately, all biomarkers with a Benjamini–Hochberg adjusted *p*-value less than 0.05 in the univariable models were entered into a multiple-biomarker model adjusted for all aforementioned confounders.

As re-intervention might influence the relationship between biomarker and sac volume, a sensitivity analysis was performed in the group of patients after EVAR, wherein patients with a re-intervention between the initial EVAR surgery (40 participants) and the current measurements were excluded. Similarly, patients who initially underwent EVAR for ruptured AAA might portray different biomarker profiles in relation to post-EVAR volume and were also excluded in this sensitivity analysis.

Although above-described cross-sectional analyses on sac volume serve as a pilot study for the prospective, longitudinal investigation of biomarkers in relation to sac volume, which is yet to follow, we recognize that sac growth or shrinkage after surgery is an important outcome.^4^ Considering the available retrospective data on sac diameter, we repeated the analyses in the EVAR group with sac growth, based on diameter, as the outcome. Sac growth was calculated as the difference between current maximal diameter and the maximal diameter at the time of the index surgery, both as measured by the radiologist.

Lastly, differences in biomarker levels between watchful waiting patients and patients after EVAR were assessed by linear regression with Benjamini–Hochberg multiple testing correction. Results are presented as the mean difference in Z-score protein abundance.

Statistical analysis was conducted using IBM SPSS Statistics (version 25.0; IBM Corp., Armonk, NY, USA) and R (4.0.3; R Foundation for Statistical Computing, Vienna, Austria), using packages NbClust^18^ and fpc.^19^ We corrected the biomarker regression analyses for multiple testing by using Benjamini–Hochberg adjusted *p*-values.^20^ Otherwise, *p* < 0.05 was considered statistically significant. All tests were two-tailed.

## Results

Laboratory analysis was performed in 316 patients. The biomarker values of three patients contained a large proportion of outliers (Z-score below −3 or above 3 in > 25% of the measured biomarkers) and these patients were omitted from current analyses (supplemental Figure S1B). In total, 91 cardiovascular disease-related biomarkers were explored in 313 patients. Thus, the current investigation comprised 110 watchful waiting patients and 203 EVAR patients.

Clinical characteristics at baseline are presented in Table 1 (89% men, mean age of 72 ± 7 years in the watchful waiting group; 92% men, 74 ± 8 years in the EVAR group). Patients were predominantly current or former smokers with a high prevalence of cardiovascular risk factors such as hypertension. The biomarker profiles of watchful waiting and EVAR patients were notably different, as reflected by the 29 significantly different protein levels between the groups (supplemental Figure S3).

**Table 1.** Clinical characteristics of watchful waiting and EVAR patients.

	Watchful Waiting	EVAR
	*n* = 110	*n* = 203
Age	71.8 ± 7.1	74.3 ± 7.6
Men	98 (89.1)	187 (92.1)
BMI	27.7 ± 3.9	26.8 ± 3.8
*Medical history*		
Coronary heart disease^a^	41 (37.3)	69 (34.0)
Heart failure	8 (7.3)	13 (6.4)
Hypertension	78 (70.9)	162 (79.8)
Cerebrovascular disease	16 (14.5)	38 (18.7)
Diabetes mellitus	27 (24.5)	35 (17.2)
Peripheral artery occlusive disease	24 (21.8)	28 (13.8)
Chronic obstructive pulmonary disease	20 (18.2)	48 (23.6)
Smoking		
Never	3 (2.7)	10 (4.9)
Current	35 (31.8)	53 (26.1)
Former	72 (65.5)	140 (69.0)
*Medication use*		
Antiplatelet	76 (69.1)	158 (77.8)
DOAC	6 (5.5)	9 (4.4)
Coumarin	12 (10.9)	25 (12.3)
Beta-blocker	50 (45.5)	116 (57.1)
ACE inhibitor	31 (28.2)	60 (29.6)
Angiotensin II receptor antagonist	33 (30.0)	58 (28.6)
Thiazide diuretic	21 (19.1)	41 (20.2)
Lipid-lowering drug	85 (77.3)	167 (82.3)
*Aneurysm-related information*		
Anatomical classification AAA – infrarenal	104 (94.5)	181 (89.2)
Maximal diameter AAA (mm)	50.0 (46.0–53.0)	55.0 (45.0–64.0)
AAA/sac volume (mL)	106.1 (93.4–129.1)	146.8 (104.1–209.0)
Concurrent iliac artery aneurysm	18 (16.4)	56 (27.6)
Familial AAA^b^	25 (22.7)	51 (25.1)
*Surgery-related information*		
Type of index surgery		
Primary EVAR		171 (84.2)
Re-intervention		32 (15.8)
Months between index surgery and blood sample collection		38.2 (9.1–68.8)
Months between index surgery and the CT scan		38.0 (8.7–67.8)
AAA ruptured at admission		14 (6.9)
ASA classification		
ASA I or II		81 (39.9)
ASA III, IV, or IV		100 (49.3)
Unknown		22 (10.8)

Variables with a normal distribution are presented as mean ± SD, whereas nonnormally distributed continuous variables are expressed as median (25th to 75th percentile). Categorical variables are expressed as count (percentage).

a Coronary heart disease: history of myocardial infarction, and/or percutaneous coronary intervention, and/or coronary artery bypass grafting.

b Familial AAA: defined as at least one first-degree relative affected with aortic aneurysm, based on anamnestic information.

AAA, abdominal aortic aneurysm; ACE, angiotensin-converting enzyme; ASA classification, physical status classification system by the American Society of Anesthesiologists; BMI, body mass index; CT, computed tomography; EVAR, endovascular aneurysm repair; DOAC, direct oral anticoagulant.

The single measurement intraclass correlation coefficient for the volume measurements was 0.997 for the intraobserver variability and 0.998 for the interobserver variability. The mean difference for the intra- and interobserver variability were 0.0852 mL (SD 4.777) and −0.1293 mL (SD 5.227), respectively.

In the watchful waiting patients, CT scans showed a maximum AAA diameter with a median of 50 (25th to 75th percentile: 46.0–53.0) mm, and median aneurysm volume was 106.1 (93.4–129.1) mL. For EVAR patients, we measured a maximum aneurysm sac diameter of 55.0 (45.0–64.0) mm and a volume of 146.8 (104.1–209.0) mL (Table 1). Median time between EVAR and CT scan, corresponding with the interval between EVAR and biomarker measurement, was 38.0 (8.7–67.8) months (Table 1). There were 43 patients who had early blood sampling within 90 days following EVAR, compared to the rest of the participants whose blood sampling was fairly evenly distributed over the remaining time period after EVAR (supplemental Figure S4). In the 43 patients with blood sampling < 90 days after EVAR, only one biomarker, myoglobin, showed a significantly different level, compared to the 160 patients with sampling > 90 days after EVAR (supplemental Figure S5).

### Cluster analyses

Cluster analysis based solely on biomarker profile resulted in two different patient clusters (cluster 1 and cluster 2) for both the watchful waiting and the EVAR group. Supplemental Figure S6 and 5B contain heatmaps visualizing the clusters, with higher biomarker concentrations in cluster 2 of both groups, as depicted by the red coloring. This was especially pronounced in the EVAR group (supplemental Figure S6B). In online supplemental Tables S2 and S3, biomarker concentrations in NPX values are presented for the total group and each of the two clusters, for watchful waiting patients and EVAR patients, respectively. In both the watchful waiting and EVAR groups, patients in cluster 2 had higher concentrations of the vast majority of biomarkers compared to cluster 1. Patients in the high biomarker cluster of the watchful waiting group were older and more frequently used coumarin and thiazide diuretics compared to those in the low biomarker cluster (supplemental Table S4). Patients in the high biomarker cluster of the EVAR group were older and were more likely to use beta-blockers compared to those in the low biomarker cluster (supplemental Table S5). No difference in maximal diameter or AAA/sac volume could be observed between the clusters. Pathway analysis did not reveal any pathways underlying the differently expressed proteins among watchful waiting or EVAR groups.

### Linear regression analyses

With the cluster analyses, we examined all biomarkers in a comprehensive manner. Subsequently, we examined associations of individual biomarkers with AAA or sac volume. Figure 1 shows the results of univariable linear regression for the watchful waiting patients. Urokinase-type plasminogen activator (uPA) showed a borderline significant association with AAA volume with a β (95% CI) of −0.097 (−0.151, −0.043) log_e_ mL in AAA volume per SD difference of the biomarker NPX value, after correction for multiple testing. When adjusting the association of uPA and AAA volume for age, sex, and BSA (model A), and subsequently for cardiovascular disease-related characteristics (model C), the effect size remained similar, respectively a change of −0.094 (−0.150, −0.038) and −0.092 (−0.148, −0.036) log_e_ mL in AAA volume per SD increase in biomarker level. These associations, however, lost significance after correcting for multiple testing. No multiple-biomarker linear regression models were applied for watchful waiting patients as none of the biomarkers reached statistical significance in univariable models after adjustment for multiple testing.

**Figure 2.**
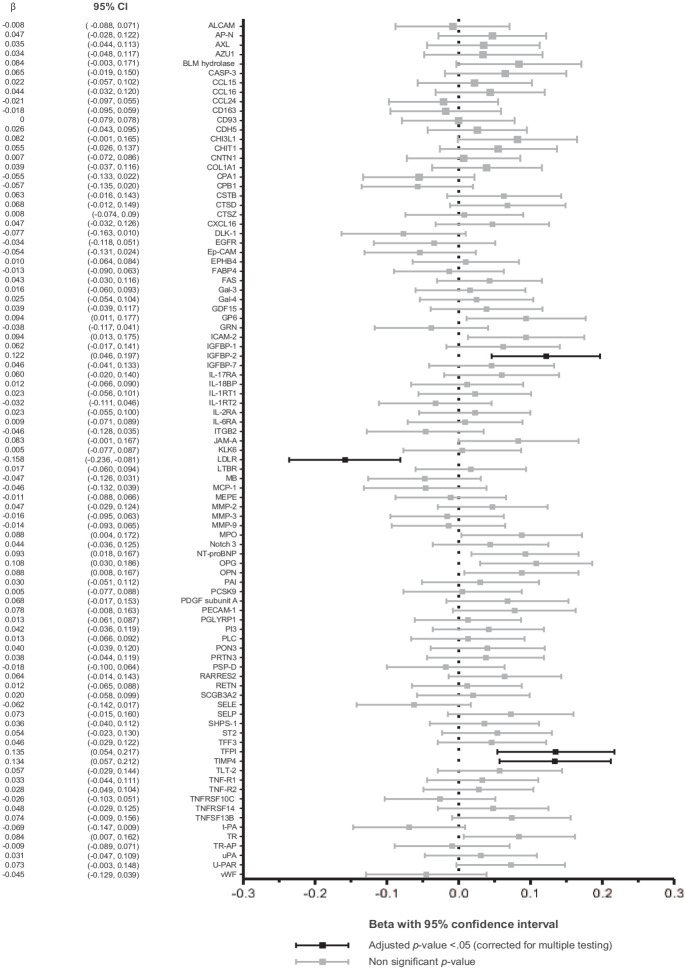
Association of biomarkers with aneurysm sac volume in patients after EVAR. This figure represents the results of univariable linear regression where we examined associations of individual biomarkers with sac volume for patients after EVAR. Results are presented as the mean effect (β) with 95% CI of a 1 SD difference of the biomarker on sac volume (expressed as natural log mL). EVAR, endovascular aneurysm repair.

In the patients after EVAR surgery, several biomarkers showed significant associations with sac volume (Figure 2). One SD difference in low-density lipoprotein receptor (LDLR) was related with a mean difference in sac volume of −0.158 (95% CI: −0.236, −0.081) log_e_ mL. The mean effects on sac volume per SD difference were 0.122 (0.046, 0.197) for insulin-like growth factor-binding protein 2 (IGFBP-2), 0.135 (0.054, 0.217) for tissue factor pathway inhibitor (TFPI), and 0.134 (0.057, 0.212) for metalloproteinase inhibitor 4 (TIMP4).

After adjustment for age, sex, and BSA (A), time between EVAR and blood sampling (B), and additionally cardiovascular disease-related characteristics (C), all four biomarkers remained significantly associated with sac volume (Table 2). The strongest associations in model C were observed for LDLR and TFPI, with a mean effect on sac volume per SD difference of −0.128 (95% CI −0.212, −0.044) log_e_ mL and 0.139 (0.049, 0.229) log_e_ mL, respectively. In an additional multiple-biomarker model including the four biomarkers and the before-mentioned covariates, the statistical significance of the relation with sac volume remained for LDLR (−0.173 [−0.262, −0.084], adjusted *p*-value 0.001) followed by TFPI (0.180 [0.074, 0.287], *p*-value 0.006), whereas TIMP4 (0.053 [−0.039, 0.144], *p*-value 0.62) and IGFBP-2 (−0.029 [−0.134, 0.076], *p*-value 0.79) lost significance. A sensitivity analysis showed that the results were robust to the exclusion of patients with a history of rupture or re-intervention (supplemental Table S6). When repeating analyses for sac growth based on diameter measurements, only LDLR was found to be significantly associated with the outcome measure. After adjusting for multiple covariates (C), an increase of 1 SD in LDLR was associated with a reduction of 2.527 mm in diameter since the index surgery (95% CI 0.569, 4.486, *p*-value 0.012) (supplemental Table S7 and Figure S7).

**Table 2.** Association of biomarkers with aneurysm sac volume in EVAR patients – adjusted for confounders.

	**A**			**B**			**C**		
	β	95% CI	Adj. *p*-value	β	95% CI	Adj. *p*-value	β	95% CI	Adj. *p*-value
IGFBP2	0.133	(0.047, 0.219)	0.003	0.099	(0.013, 0.186)	0.025	0.103	(0.012, 0.194)	0.027
LDLR	−0.157	(−0.238, −0.077)	< 0.001	−0.125	(−0.207, −0.044)	0.011	−0.128	(−0.212, −0.044)	0.006
TFPI	0.135	(0.052, 0.218)	0.002	0.113	(0.031, 0.195)	0.011	0.139	(0.049, 0.229)	0.006
TIMP4	0.135	(0.051, 0.219)	0.002	0.113	(0.030, 0.195)	0.011	0.110	(0.023, 0.197)	0.018

Linear regression analysis was applied to examine associations of individual biomarkers with sac volume.

**A**: Biomarkers with an adjusted *p* < 0.05 in univariable linear regression were adjusted for age, sex, and body surface area.

**B**: Additionally, time between EVAR surgery and study visit was also added to the model.

**C**: Finally, additional predefined variables were added: history of coronary heart disease, hypertension, diabetes mellitus, smoking status, peripheral artery occlusive disease, antiplatelet therapy, lipid-lowering drug therapy, and familial abdominal aortic aneurysm.

Results are presented as the mean effect with 95% CI of a 1 SD difference of the biomarker on sac volume (expressed as loge mL).

We corrected for multiple testing concluding significance with a Benjamini–Hochberg adjusted p < 0.05.

adj, adjusted; β, regression coefficient; EVAR, endovascular aneurysm repair; IGFBP-2, insulin-like growth factor-binding protein 2; LDLR, low-density lipoprotein receptor; TFPI, tissue factor pathway inhibitor; TIMP4, metalloproteinase inhibitor 4.

## Discussion

At present, monitoring strategies in patients with AAA are based on imaging techniques and leave room for improvement for timely identification of patients at high risk for adverse events. Circulating biomarkers with prognostic abilities for aneurysm or sac growth carry potential to improve and personalize monitoring strategies. We show that in patients after EVAR procedure, LDLR, TFPI, TIMP4, and IGFBP-2 are associated with volume of the aneurysm sac. The inverse association of LDLR and the positive association of TFPI persist after adjustment for clinical characteristics at baseline and other biomarkers, suggesting potential independent roles for circulating LDLR and TFPI as biomarkers for sac volume in patients after EVAR. LDLR might be of special interest as a candidate biomarker in patients following EVAR, as it showed to be inversely related to sac growth. Furthermore, we demonstrate the presence of subgroups of patients with high levels of the majority of the cardiovascular disease-related proteins, but without differences in AAA diameter and volume between the clusters. Clinical characteristics (except age) were also similar between clusters. Although these subgroups do not have particular AAA characteristics, their presence suggests that accounting for potential simultaneous occurrence of both AAA and other cardiovascular diseases may be warranted in further analyses, and we did so by adjusting for clinical characteristics. Altogether, LDLR and several other markers are of interest in future research concerning risk stratification, and the identified clusters emphasize the intertwined relationship between AAA and cardiovascular disease.

To our knowledge, we are the first to describe the associations of a broad range of circulating biomarkers related to cardiovascular disease with AAA volume in both patients under surveillance and patients after EVAR. Our study differs from previous investigations, not only by the use of aneurysm volume as the outcome measure (which is a reliable measurement methodology^15,16^) but also by the considerable number of patients in both the pre- and postoperative stage of the disease, and the prespecified cohort study design.

LDLR regulates cholesterol homeostasis by endocytosis of LDL, and most circulating LDL is cleared by hepatic LDLR.^21^ In our study, LDLR was found to be inversely associated with aneurysm sac volume in patients after EVAR procedure. LDLR plays an important role in the cholesterol-lowering effect of statins.^22^ The association remained strongly present even after adjustment for lipid-lowering drug use, encouraging further investigation of LDLR in relation to AAA. The interest in LDLR is further strengthened by the findings of genome-wide association studies, where a variant in LDLR was identified that was associated with AAA, independently of LDL-cholesterol levels.^23^ Additionally, the importance of LDLR in patients after EVAR was substantiated by our sensitivity analysis demonstrating its association with sac shrinkage, corrected for time since EVAR. These results should, however, be interpreted with caution, as they reflect on retrospectively assessed sac shrinkage, averaged over time. Moreover, the relationship between the transmembrane LDLR protein and its shedded circulating domain has not been fully established.^24^

Although not clearly understood yet, the intraluminal thrombus is suggested to be biochemically active in AAA growth by inducing fibrinolytic activity and proteolysis which might weaken the artery wall.^25^ Especially in the light of this process, TFPI, which we found is associated with sac volume after EVAR, might be of future interest. Additionally, TFPI is also involved in the regulation of coagulation^26^ and previously, plasma TFPI correlated with several hemostatic factors and with AAA diameter in preoperative patients.^27^ Previous authors have described a decrease in thrombin activation months after EVAR, compared to the preoperative state, but the relationship with sac size has not yet been investigated.^28^ Our results might indicate endothelial activation or a decrease in thrombin activation proportional to the sac size, months to years after EVAR. Future studies taking into account the TFPI isoform might be able to elucidate exact biological mechanisms and cell-types involved in post-EVAR sac dynamics.^29^

Matrix metalloproteinases have been linked to the pathogenesis of AAA, due to their role as proteolytic enzyme in remodeling of the extracellular matrix of the aortic wall.^30^ TIMP4 was significantly associated with sac volume after EVAR in our study, which has not yet been shown before. This protein is a tissue inhibitor of the typically cardiovascular metalloproteinases 2 and 9, whose elevated levels play a key role in elastic fiber degradation in AAA.^31^ In line with the results of our multiple marker model, Hu et al. demonstrated the interactive process of LDLR and TIMP4, as well as their dysregulatory effects on a variety of metalloproteinases, in atherosclerotic AAA.^32^ In contrast to TIMP1 and -2, TIMP4 is more specific to the abdominal aorta.^30,32,33^ This could explain why we could relate TIMP4 to post-EVAR sac size, contrary to previous authors that investigated TIMP1 in AAA.^9^

The final biomarker we found to be associated with sac volume after EVAR was IGFBP-2, a binding protein primarily known for its modulating capacity on especially insulin-like-growth factor-1.^34^ This protein has been suggested to be involved in the process of atherosclerosis^34^ and as a biomarker in other cardiovascular diseases like heart failure^35^ and diabetes mellitus.^36^ Together with that of TIMP4, the association of IGFBP2 with post-EVAR volume disappeared when combining all biomarkers in one model. This might indicate common mechanisms with regard to sac volume between LDLR, TIMP4, and IGFBP2; their biological interaction relating to metabolic dysregulation further strengthens these findings.^30–32,37^ Future research into post-EVAR sac behavior and biomarkers should elucidate the role of such processes in long-term risk and survival.

In watchful waiting patients, we found a borderline significant association between uPA and aneurysm volume. In several murine models, uPA was investigated as a possible mediator of proteolysis and inflammation in AAA^38^ and uPA plays a pivotal role in extracellular matrix degradation.^39^ The exact mechanisms, however, to stimulate AAA are not yet defined.

Previously, in a case–control design within a population-based screening cohort of patients with AAA, Memon and colleagues identified a significant increase of 21 biomarkers of proteolysis, oxidative stress, lipid metabolism, and inflammation that was associated with AAA diameter and growth, as well as a decrease of paraoxonase-3.^40^ We did not find an overlap between the biomarkers identified in that study and our results in watchful waiting patients, which may in part be explained by the different study design.

Cluster analysis based on biomarker levels resulted in two different patient clusters for both groups. In each group, one cluster had higher levels of the majority of biomarkers compared to the other cluster. The currently investigated biomarkers were all cardiovascular disease-related. Given the overlap in biological processes and risk factors involved in both AAA and cardiovascular disease, and overlap in the occurrence of AAA and such diseases, the elevated levels might reflect the atherosclerotic process and other cardiovascular diseases as well as AAA. Correspondingly, no differences in aortic diameter and volume were found between clusters, and baseline characteristics (except age) were similar. Still, whether the relationship between atherosclerosis and AAA is causal or merely due to shared risk factors remains unknown.^41^ In our linear regression models we accounted for potential overlapping occurrence by adjusting for clinical characteristics and medication use.

Some aspects of our study warrant consideration. First, though the study design of BIOMArCS-AAA was prospective with repeated measurements of aortic volume and blood sampling, the current cross-sectional investigation comprised one sample per patient. As such, the current results are regarded as a pilot for further in-depth research, wherein the role of the currently identified biomarkers could be further elucidated. In the future, repeated measurements will allow us to associate changes in biomarker levels with aneurysm growth, to identify high-risk patients in need of extra surveillance. Second, due to the natural course of the disease, variability in aortic volume is considerably smaller in watchful waiting patients (106.1 [93.4, 129.1] mL) as compared to patients after EVAR surgery (146.8 [104.1, 209.0] mL). Together with a lower number of patients, this could partially explain the difficulty in finding an association with biomarkers in watchful waiting patients. Third, the vast majority of included patients were men, as typically seen in AAA-related studies. Lastly, the targeted multiplex biomarker panel allowed only limited and prespecified coverage of the human proteome; the current panel did not include D-dimer, one of the candidate markers that has been associated with AAA size and growth in several studies so far.^42^ Although D-dimer could be useful in AAA screening, its weak to moderate correlations with AAA size described by the few large studies performed thus far, should warrant further exploration into alternatives.^42,43^ All the more when a considerable part of the association between AAA size and D-dimer can be attributable to variations in intraluminal thrombus size.^42^ Nevertheless, a comparison with D-dimer could have provided some additional insight into the relevance of our findings.

## Conclusion

The current study showed an association between LDLR, TFPI, TIMP4, and IGFBP-2 and sac volume in patients after EVAR, independent of cardiovascular risk factors. The subgroups of patients with high levels of cardiovascular disease-related biomarkers emphasize the intertwined relationship between AAA and other cardiovascular diseases. After completion of follow-up, the longitudinal data from our cohort will be used to investigate whether the currently implicated markers are associated with aneurysm growth and adverse cardiovascular events, and clarify their role in risk stratification and increasingly personalized surveillance programs for patients with AAA.

## Supplemental Material

sj-pdf-1-vmj-10.1177_1358863X231181159 – Supplemental material for Circulating biomarkers of cardiovascular disease are related to aneurysm volume in abdominal aortic aneurysm Click here for additional data file. Supplemental material, sj-pdf-1-vmj-10.1177_1358863X231181159 for Circulating biomarkers of cardiovascular disease are related to aneurysm volume in abdominal aortic aneurysm by Elke Bouwens, Alexander Vanmaele, Sanne E Hoeks, Hence JM Verhagen, Bram Fioole, Adriaan Moelker, Sander ten Raa, Burhan Hussain, José Oliveira-Pinto, Frederico Bastos Gonçalves, Arne S Ijpma, Imo E Hoefer, Felix van Lier, K Martijn Akkerhuis, Danielle F Majoor-Krakauer, Eric Boersma and Isabella Kardys in Vascular Medicine

sj-pdf-2-vmj-10.1177_1358863X231181159 – Supplemental material for Circulating biomarkers of cardiovascular disease are related to aneurysm volume in abdominal aortic aneurysm Click here for additional data file. Supplemental material, sj-pdf-2-vmj-10.1177_1358863X231181159 for Circulating biomarkers of cardiovascular disease are related to aneurysm volume in abdominal aortic aneurysm by Elke Bouwens, Alexander Vanmaele, Sanne E Hoeks, Hence JM Verhagen, Bram Fioole, Adriaan Moelker, Sander ten Raa, Burhan Hussain, José Oliveira-Pinto, Frederico Bastos Gonçalves, Arne S Ijpma, Imo E Hoefer, Felix van Lier, K Martijn Akkerhuis, Danielle F Majoor-Krakauer, Eric Boersma and Isabella Kardys in Vascular Medicine

sj-pdf-3-vmj-10.1177_1358863X231181159 – Supplemental material for Circulating biomarkers of cardiovascular disease are related to aneurysm volume in abdominal aortic aneurysm Click here for additional data file. Supplemental material, sj-pdf-3-vmj-10.1177_1358863X231181159 for Circulating biomarkers of cardiovascular disease are related to aneurysm volume in abdominal aortic aneurysm by Elke Bouwens, Alexander Vanmaele, Sanne E Hoeks, Hence JM Verhagen, Bram Fioole, Adriaan Moelker, Sander ten Raa, Burhan Hussain, José Oliveira-Pinto, Frederico Bastos Gonçalves, Arne S Ijpma, Imo E Hoefer, Felix van Lier, K Martijn Akkerhuis, Danielle F Majoor-Krakauer, Eric Boersma and Isabella Kardys in Vascular Medicine
