# Supplementary material for: Circulating biomarkers of cardiovascular disease are related to aneurysm volume in abdominal aortic aneurysm
Source: Vasc Med. 2023 Jul 3;28(5):433–42. doi: 10.1177/1358863X231181159 (PMC10559648; doi:10.1177/1358863X231181159)
Supplement: sj-pdf-1-vmj-10.1177_1358863X231181159 – Supplemental material for Circulating biomarkers of cardiovascular disease are related to aneurysm volume in abdominal aortic aneurysm [file sj-pdf-1-vmj-10.1177_1358863X231181159.pdf]

Table S1. Cardiovascular related biomarkers included in the Cardiovascular III panel

Abbreviation	Full biomarker name	Abbreviation	Full biomarker name
ALCAM	CD166 antigen	LDLR	Low-density lipoprotein receptor
AP-N	Aminopeptidase N	LTBR	Lymphotoxin-beta receptor
AXL	Tyrosine-protein kinase receptor UFO	MB	Myoglobin
AZU1	Azurocidin	MCP-1	Monocyte chemotactic protein 1
BLM hydrolase	Bleomycin hydrolase	MEPE	Matrix extracellular phosphoglycoprotein
CASP-3	Caspase-3	MMP-2	Matrix metalloproteinase-2
CCL15	C-C motif chemokine 15	MMP-3	Matrix metalloproteinase-3
CCL16	C-C motif chemokine 16	MMP-9	Matrix metalloproteinase-9
CCL24	C-C motif chemokine 24	MPO	Myeloperoxidase
CD163	Scavenger receptor cysteine-rich type 1 protein M130	Notch 3	Neurogenic locus notch homolog protein 3
CD93	Complement component C1q receptor	NT-proBNP	N-terminal prohormone brain natriuretic peptide
CDH5	Cadherin-5	OPG	Osteoprotegerin
CHI3L1	Chitinase-3-like protein 1	OPN	Osteopontin
CHIT1	Chitotriosidase-1	PAI	Plasminogen activator inhibitor 1
CNTN1	Contactin-1	PCSK9	Proprotein convertase subtilisin/kexin type 9
COL1A1	Collagen alpha-1(I) chain	PDGF subunit A	Platelet-derived growth factor subunit A
CPA1	Carboxypeptidase A1	PECAM-1	Platelet endothelial cell adhesion molecule
CPB1	Carboxypeptidase B	PGLYRP1	Peptidoglycan recognition protein 1
CSTB	Cystatin-B	PI3	Elafin
CTSD	Cathepsin D	PLC	Perlecan
CTSZ	Cathepsin Z	PON3	Paraoxonase 3
CXCL16	C-X-C motif chemokine 16	PRTN3	Myeloblastin
DLK-1	Protein delta homolog 1	PSP-D	Pulmonary surfactant-associated protein D
EGFR	Epidermal growth factor receptor	RARRES2	Retinoic acid receptor responder protein 2
Ep-CAM	Epithelial cell adhesion molecule	RETN	Resistin
EPHB4	Ephrin type-B receptor 4	SCGB3A2	Secretoglobin family 3A member 2
FABP4	Fatty acid-binding protein, adipocyte	SELE	E-selectin
FAS	Tumor necrosis factor receptor superfamily member 6	SELP	P-selectin
Gal-3	Galectin-3	SHPS-1	Tyrosine-protein phosphatase nonreceptor type substrate 1
Gal-4	Galectin-4	ST2	ST2 protein
GDF15	Growth/differentiation factor 15	TFF3	Trefoil factor 3
GP6	Platelet glycoprotein VI	TFPI	Tissue factor pathway inhibitor
GRN	Granulins	TIMP4	Metalloproteinase inhibitor 4
ICAM-2	Intercellular adhesion molecule 2	TLT-2	Trem-like transcript 2 protein
IGFBP-1	Insulin-like growth factor-binding protein 1	TNF-R1	Tumor necrosis factor receptor 1
IGFBP-2	Insulin-like Growth Factor-Binding Protein 2	TNF-R2	Tumor necrosis factor receptor 2
IGFBP-7	Insulin-like growth factor-binding protein 7	TNFRSF10C	Tumor necrosis factor receptor superfamily member 10C
IL-17RA	Interleukin-17 receptor A	TNFRSF14	Tumor necrosis factor receptor superfamily member 14
IL-18BP	Interleukin-18-binding protein	TNFSF13B	Tumor necrosis factor ligand superfamily member 13B
IL-1RT1	Interleukin-1 receptor type 1	t-PA	Tissue-type plasminogen activator
IL-1RT2	Interleukin-1 receptor type 2	TR	Transferrin receptor protein 1
IL-2RA	Interleukin-2 receptor subunit alpha	TR-AP	Tartrate-resistant acid phosphatase type 5
IL-6RA	Interleukin-6 receptor subunit alpha	uPA	Urokinase-type plasminogen activator
ITGB2	Integrin beta-2	U-PAR	Urokinase plasminogen activator surface receptor
JAM-A	Junctional adhesion molecule A	vWF	von Willebrand factor
KLK6	Kallikrein-6		

Table S2. Biomarker concentrations (in NPX value) in watchful waiting patients according to clusters

	Watchful waiting n=110	Cluster 1 n=62	Cluster 2 n=48	Adj *p*-value
ALCAM	6.365 (6.219 - 6.583)	6.251 (6.149 - 6.367)	6.596 (6.442 - 6.695)	<.001
AP-N	4.872 (4.714 - 5.064)	4.870 (4.701 - 5.031)	4.929 (4.768 - 5.081)	.35
AXL	7.546 (7.311 - 7.746)	7.400 (7.187 - 7.567)	7.692 (7.541 - 7.928)	<.001
AZU1	2.349 (2.098 - 2.565)	2.225 (1.978 - 2.454)	2.460 (2.274 - 2.632)	<.001
BLM hydrolase	2.834 (2.608 - 3.183)	2.777 (2.568 - 3.047)	3.038 (2.754 - 3.316)	.013
CASP-3*	8.260 (7.424 - 8.926)	8.192 (7.285 - 8.788)	8.391 (7.654 - 9.039)	.22
CCL15	6.960 (6.669 - 7.236)	6.724 (6.546 - 6.925)	7.160 (7.056 - 7.483)	<.001
CCL16	6.075 (5.708 - 6.404)	5.927 (5.512 - 6.143)	6.394 (6.003 - 6.599)	<.001
CCL24	5.170 (4.530 - 5.783)	5.321 (4.493 - 5.869)	5.091 (4.584 - 5.648)	.68
CD163	7.059 (6.839 - 7.363)	6.957 (6.763 - 7.162)	7.236 (7.026 - 7.525)	<.001
CD93	10.311 (9.997 - 10.504)	10.118 (9.914 - 10.316)	10.494 (10.344 - 10.660)	<.001
CDH5	2.727 (2.557 - 2.923)	2.651 (2.491 - 2.764)	2.859 (2.717 - 3.069)	<.001
CHI3L1	6.079 (5.356 - 6.795)	5.689 (5.119 - 6.200)	6.735 (5.977 - 7.259)	<.001
CHIT1	4.334 (3.610 - 4.922)	4.104 (3.326 - 4.579)	4.814 (3.961 - 5.169)	.003
CNTN1	3.446 (3.222 - 3.592)	3.360 (3.123 - 3.563)	3.530 (3.293 - 3.651)	.012
COL1A1	1.714 (1.497 - 1.908)	1.641 (1.427 - 1.844)	1.795 (1.593 - 2.031)	.01
CPA1	4.634 (4.152 - 5.150)	4.516 (4.008 - 5.051)	4.851 (4.324 - 5.239)	.16
CPB1*	5.219 (4.786 - 5.746)	5.147 (4.623 - 5.634)	5.396 (4.884 - 5.883)	.083
CSTB	4.548 (4.158 - 4.990)	4.296 (4.008 - 4.506)	5.019 (4.729 - 5.288)	<.001
CTSD	2.954 (2.737 - 3.321)	2.807 (2.607 - 3.082)	3.151 (2.859 - 3.406)	<.001
CTSZ	4.016 (3.717 - 4.229)	3.765 (3.560 - 4.031)	4.221 (4.097 - 4.392)	<.001
CXCL16	5.212 (4.995 - 5.448)	5.099 (4.929 - 5.257)	5.410 (5.210 - 5.592)	<.001
DLK-1	5.270 (4.740 - 5.772)	5.027 (4.513 - 5.439)	5.496 (5.258 - 5.942)	<.001
EGFR	1.654 (1.549 - 1.751)	1.642 (1.554 - 1.730)	1.680 (1.547 - 1.764)	.36
Ep-CAM	4.527 (4.212 - 5.317)	4.521 (4.125 - 5.260)	4.532 (4.273 - 5.351)	.40
EPHB4*	4.690 (4.513 - 4.929)	4.546 (4.386 - 4.683)	4.996 (4.830 - 5.154)	<.001
FABP4	4.428 (4.008 - 5.091)	4.129 (3.686 - 4.478)	5.039 (4.492 - 5.540)	<.001
FAS	4.866 (4.642 - 5.071)	4.710 (4.567 - 4.854)	5.120 (4.922 - 5.335)	<.001
Gal-3	4.164 (3.924 - 4.372)	4.030 (3.878 - 4.199)	4.369 (4.178 - 4.500)	<.001
Gal-4	3.692 (3.327 - 3.977)	3.525 (3.187 - 3.771)	3.899 (3.664 - 4.049)	<.001
GDF15	5.218 (4.760 - 5.628)	4.813 (4.513 - 5.185)	5.509 (5.297 - 5.905)	<.001
GP6*	2.882 (2.307 - 3.234)	2.759 (2.159 - 3.225)	2.972 (2.431 - 3.250)	.16
GRN	4.345 (4.167 - 4.501)	4.237 (4.044 - 4.377)	4.482 (4.334 - 4.607)	<.001
ICAM-2	4.553 (4.361 - 4.740)	4.491 (4.241 - 4.623)	4.630 (4.415 - 4.932)	.001
IGFBP-1	4.596 (3.617 - 5.453)	4.458 (3.252 - 5.020)	5.072 (4.302 - 5.764)	.001
IGFBP-2	7.925 (7.430 - 8.491)	7.662 (7.230 - 8.183)	8.227 (7.693 - 8.925)	.001
IGFBP-7	7.907 (7.678 - 8.195)	7.731 (7.502 - 7.897)	8.193 (7.985 - 8.290)	<.001
IL-17RA	3.202 (2.919 - 3.464)	3.049 (2.854 - 3.309)	3.433 (3.168 - 3.746)	<.001
IL-18BP*	5.621 (5.363 - 5.908)	5.436 (5.266 - 5.600)	5.961 (5.757 - 6.121)	<.001
IL-1RT1	4.869 (4.672 - 5.039)	4.721 (4.579 - 4.883)	5.051 (4.879 - 5.229)	<.001
IL-1RT2	4.920 (4.705 - 5.103)	4.895 (4.686 - 5.069)	4.982 (4.760 - 5.178)	.15
IL-2RA	3.943 (3.665 - 4.235)	3.802 (3.368 - 3.996)	4.127 (3.864 - 4.483)	<.001
IL-6RA	11.416 (11.114 - 11.621)	11.326 (10.968 - 11.486)	11.557 (11.261 - 11.831)	<.001
ITGB2	4.565 (4.320 - 4.825)	4.463 (4.252 - 4.703)	4.735 (4.546 - 4.896)	<.001
JAM-A*	6.299 (5.767 - 7.028)	6.199 (5.582 - 6.715)	6.598 (5.888 - 7.078)	.037
KLK6	3.538 (3.270 - 3.811)	3.395 (3.196 - 3.571)	3.779 (3.535 - 4.154)	<.001
LDLR	3.908 (3.624 - 4.246)	3.864 (3.618 - 4.214)	4.046 (3.694 - 4.313)	.16
LTBR*	3.126 (2.908 - 3.387)	2.927 (2.756 - 3.102)	3.403 (3.261 - 3.690)	<.001

MB	6.275 (5.849 - 6.702)	6.098 (5.696 - 6.512)	6.463 (6.161 - 6.927)	.004
MCP-1	4.059 (3.799 - 4.203)	3.910 (3.773 - 4.082)	4.147 (4.057 - 4.320)	<.001
MEPE	3.782 (3.517 - 4.131)	3.644 (3.356 - 3.820)	4.138 (3.779 - 4.395)	<.001
MMP-2	2.264 (2.113 - 2.547)	2.203 (2.046 - 2.361)	2.404 (2.246 - 2.641)	<.001
MMP-3	5.785 (5.337 - 6.266)	5.688 (5.172 - 5.921)	6.167 (5.669 - 6.524)	<.001
MMP-9	4.177 (3.487 - 4.391)	4.020 (3.414 - 4.377)	4.261 (3.587 - 4.397)	.35
MPO	2.199 (1.967 - 2.461)	2.074 (1.832 - 2.284)	2.345 (2.213 - 2.588)	<.001
Notch 3	4.353 (4.087 - 4.569)	4.205 (3.966 - 4.409)	4.522 (4.318 - 4.849)	<.001
NT-proBNP	3.510 (2.691 - 4.418)	3.089 (2.517 - 3.787)	3.846 (3.253 - 4.961)	.002
OPG	3.060 (2.870 - 3.350)	2.963 (2.711 - 3.119)	3.291 (2.996 - 3.524)	<.001
OPN	7.263 (6.886 - 7.622)	6.937 (6.727 - 7.268)	7.564 (7.362 - 7.888)	<.001
PAI*	5.625 (5.150 - 5.974)	5.593 (5.150 - 5.886)	5.689 (5.152 - 6.175)	.34
PCSK9	2.183 (2.027 - 2.411)	2.149 (1.933 - 2.328)	2.317 (2.157 - 2.508)	<.001
PDGF subunit A	2.660 (2.044 - 3.099)	2.631 (2.044 - 3.050)	2.669 (2.069 - 3.132)	.34
PECAM-1*	4.613 (4.157 - 5.144)	4.511 (4.078 - 5.020)	4.858 (4.406 - 5.294)	.023
PGLYRP1	7.185 (6.948 - 7.413)	7.018 (6.810 - 7.230)	7.363 (7.171 - 7.679)	<.001
PI3	2.268 (1.973 - 2.859)	2.049 (1.775 - 2.298)	2.831 (2.318 - 3.233)	<.001
PLC*	7.442 (7.147 - 7.694)	7.186 (7.002 - 7.372)	7.721 (7.574 - 7.912)	<.001
PON3	4.578 (4.142 - 4.927)	4.622 (4.170 - 4.892)	4.556 (4.029 - 4.938)	.60
PRTN3	3.701 (3.365 - 3.954)	3.561 (3.225 - 3.799)	3.805 (3.664 - 4.004)	<.001
PSP-D	2.537 (2.129 - 3.006)	2.403 (2.076 - 2.823)	2.679 (2.290 - 3.140)	.073
RARRES2	10.909 (10.676 - 11.048)	10.730 (10.547 - 10.917)	11.032 (10.919 - 11.182)	<.001
RETN	6.341 (5.916 - 6.615)	5.975 (5.763 - 6.405)	6.553 (6.299 - 6.974)	<.001
SCGB3A2	2.176 (1.708 - 2.697)	1.985 (1.454 - 2.523)	2.473 (2.046 - 2.932)	<.001
SELE	9.602 (9.183 - 9.941)	9.464 (9.090 - 9.817)	9.715 (9.427 - 10.042)	.034
SELP*	9.658 (9.081 - 10.149)	9.503 (9.052 - 10.043)	9.917 (9.330 - 10.233)	.029
SHPS-1	3.139 (2.903 - 3.397)	3.012 (2.803 - 3.157)	3.371 (3.142 - 3.658)	<.001
ST2	6.015 (5.711 - 6.301)	5.911 (5.624 - 6.205)	6.114 (5.840 - 6.407)	.011
TFF3	5.947 (5.496 - 6.281)	5.624 (5.362 - 5.907)	6.287 (6.129 - 6.688)	<.001
TFPI	8.423 (8.257 - 8.690)	8.339 (8.126 - 8.521)	8.603 (8.374 - 8.732)	.001
TIMP4	4.109 (3.727 - 4.506)	3.946 (3.675 - 4.153)	4.452 (4.137 - 4.782)	<.001
TLT-2	4.431 (4.193 - 4.704)	4.333 (4.106 - 4.465)	4.614 (4.422 - 4.872)	<.001
TNF-R1*	5.874 (5.559 - 6.197)	5.599 (5.427 - 5.742)	6.240 (6.097 - 6.633)	<.001
TNF-R2*	4.621 (4.344 - 5.000)	4.382 (4.181 - 4.548)	5.107 (4.772 - 5.325)	<.001
TNFRSF10C	3.907 (3.543 - 4.195)	3.774 (3.405 - 3.975)	4.141 (3.749 - 4.323)	<.001
TNFRSF14	4.333 (4.081 - 4.735)	4.122 (3.969 - 4.267)	4.773 (4.524 - 4.963)	<.001
TNFSF13B	6.330 (6.107 - 6.581)	6.263 (6.057 - 6.402)	6.439 (6.295 - 6.737)	.001
t-PA	4.849 (4.423 - 5.186)	4.612 (4.265 - 4.944)	5.139 (4.846 - 5.426)	<.001
TR	4.256 (3.853 - 4.716)	4.152 (3.763 - 4.379)	4.395 (3.911 - 4.858)	.032
TR-AP	4.027 (3.796 - 4.289)	3.953 (3.797 - 4.114)	4.135 (3.804 - 4.455)	.023
uPA	4.700 (4.505 - 4.813)	4.575 (4.450 - 4.740)	4.791 (4.697 - 4.924)	<.001
U-PAR	4.628 (4.360 - 4.952)	4.393 (4.242 - 4.588)	4.955 (4.812 - 5.119)	<.001
vWF	5.661 (5.150 - 6.174)	5.419 (4.881 - 5.703)	6.180 (5.675 - 6.711)	<.001

Biomarkers are expressed in Normalized Protein eXpression (NPX), which are relative units that result from the polymerase chain reaction and which are on the log2 scale. A high NPX value equals a high biomarker concentration and, because of the log2 scale, one unit difference in NPX represents a doubling of the protein concentration. All biomarkers are expressed as median (25th - 75th percentile).

* For highly correlated biomarker pairs (Pearson's correlation coefficient >0.8), the biomarker with the highest mean correlation was excluded. Accordingly,13 biomarkers were excluded from the cluster analysis in the watchful waiting group: CASP-3, CPB1, EPHB4, GP6, IL18BP, JAM-A, LTBR, PAI, PECAM-1, PLC, SELP, TNF-R1, TNF-R2.

Table S3. Biomarker concentrations (in NPX value) in EVAR patients according to clusters

	EVAR<br>n=203	Cluster 1<br>n=136	Cluster 2<br>n=67	Adj *p*-value
ALCAM	6.416 (6.249 - 6.574)	6.335 (6.199 - 6.450)	6.607 (6.513 - 6.797)	<.001
AP-N	4.870 (4.713 - 5.076)	4.870 (4.703 - 5.035)	4.870 (4.750 - 5.140)	.18
AXL	7.585 (7.422 - 7.779)	7.494 (7.366 - 7.644)	7.824 (7.639 - 7.954)	<.001
AZU1	2.525 (2.227 - 2.886)	2.442 (2.169 - 2.733)	2.701 (2.477 - 3.013)	<.001
BLM hydrolase	3.152 (2.904 - 3.358)	3.149 (2.905 - 3.336)	3.152 (2.914 - 3.403)	.31
CASP-3*	8.779 (8.265 - 9.232)	8.786 (8.292 - 9.174)	8.661 (8.176 - 9.327)	.68
CCL15	7.083 (6.768 - 7.501)	6.905 (6.626 - 7.208)	7.540 (7.201 - 7.874)	<.001
CCL16	6.080 (5.786 - 6.420)	5.943 (5.493 - 6.210)	6.468 (6.142 - 6.772)	<.001
CCL24	5.075 (4.586 - 5.725)	5.031 (4.510 - 5.609)	5.144 (4.742 - 5.932)	.095
CD163	7.140 (6.829 - 7.394)	7.061 (6.786 - 7.348)	7.292 (7.036 - 7.513)	.001
CD93	10.271 (10.107 - 10.592)	10.175 (10.020 - 10.281)	10.724 (10.587 - 10.878)	<.001
CDH5	2.739 (2.542 - 2.937)	2.673 (2.487 - 2.832)	2.936 (2.854 - 3.154)	<.001
CHI3L1	6.241 (5.794 - 6.973)	6.058 (5.712 - 6.565)	6.860 (6.207 - 7.375)	<.001
CHIT1	4.391 (3.742 - 5.193)	4.321 (3.702 - 4.929)	4.736 (3.966 - 5.525)	.020
CNTN1	3.424 (3.249 - 3.607)	3.374 (3.198 - 3.563)	3.465 (3.359 - 3.759)	.001
COL1A1	1.758 (1.563 - 1.998)	1.685 (1.437 - 1.860)	1.965 (1.654 - 2.301)	<.001
CPA1	4.693 (4.259 - 5.231)	4.537 (4.126 - 5.070)	5.008 (4.652 - 5.562)	<.001
CPB1*	5.351 (4.902 - 5.891)	5.250 (4.779 - 5.649)	5.793 (5.242 - 6.161)	<.001
CSTB	4.900 (4.527 - 5.346)	4.697 (4.397 - 5.021)	5.431 (5.067 - 5.820)	<.001
CTSD	2.983 (2.776 - 3.280)	2.968 (2.727 - 3.208)	3.068 (2.884 - 3.388)	.014
CTSZ	3.998 (3.757 - 4.256)	3.842 (3.709 - 4.024)	4.324 (4.097 - 4.506)	<.001
CXCL16	5.201 (4.980 - 5.421)	5.088 (4.934 - 5.239)	5.487 (5.349 - 5.720)	<.001
DLK-1	5.261 (4.888 - 5.727)	5.078 (4.691 - 5.350)	5.758 (5.403 - 6.320)	<.001
EGFR	1.646 (1.555 - 1.723)	1.641 (1.544 - 1.718)	1.648 (1.574 - 1.745)	.22
Ep-CAM	4.542 (3.991 - 5.209)	4.545 (3.903 - 5.181)	4.533 (4.118 - 5.242)	.31
EPHB4*	4.705 (4.545 - 5.050)	4.599 (4.472 - 4.711)	5.230 (5.050 - 5.589)	<.001
FABP4	4.528 (4.044 - 5.172)	4.284 (3.844 - 4.683)	5.195 (4.667 - 5.899)	<.001
FAS	4.935 (4.703 - 5.163)	4.784 (4.620 - 4.988)	5.275 (5.103 - 5.506)	<.001
Gal-3	4.217 (4.015 - 4.470)	4.140 (3.924 - 4.321)	4.485 (4.216 - 4.657)	<.001
Gal-4	3.676 (3.373 - 4.033)	3.508 (3.249 - 3.892)	4.000 (3.697 - 4.156)	<.001
GDF15	5.248 (4.874 - 5.767)	4.982 (4.691 - 5.295)	5.813 (5.534 - 6.308)	<.001
GP6*	3.215 (2.870 - 3.630)	3.189 (2.800 - 3.547)	3.294 (2.921 - 3.759)	.14
GRN	4.377 (4.204 - 4.554)	4.292 (4.116 - 4.416)	4.572 (4.421 - 4.711)	<.001
ICAM-2	4.637 (4.405 - 4.892)	4.553 (4.378 - 4.787)	4.879 (4.609 - 5.058)	<.001
IGFBP-1	4.550 (3.794 - 5.480)	4.327 (3.530 - 5.197)	5.086 (4.508 - 6.143)	<.001
IGFBP-2	8.367 (7.716 - 8.932)	8.056 (7.494 - 8.526)	8.961 (8.427 - 9.383)	<.001
IGFBP-7	7.906 (7.690 - 8.143)	7.763 (7.638 - 7.908)	8.244 (8.102 - 8.535)	<.001
IL-17RA	3.341 (3.068 - 3.525)	3.297 (2.998 - 3.466)	3.398 (3.264 - 3.694)	.001
IL-18BP	5.633 (5.402 - 5.986)	5.515 (5.309 - 5.678)	6.049 (5.866 - 6.373)	<.001
IL-1RT1	4.891 (4.684 - 5.114)	4.783 (4.648 - 4.939)	5.178 (5.007 - 5.338)	<.001
IL-1RT2	4.822 (4.596 - 5.022)	4.840 (4.621 - 5.027)	4.795 (4.584 - 5.016)	.54
IL-2RA	3.987 (3.650 - 4.317)	3.785 (3.560 - 4.117)	4.448 (4.109 - 4.793)	<.001
IL-6RA	11.366 (11.025 - 11.616)	11.255 (10.957 - 11.515)	11.551 (11.253 - 11.842)	<.001
ITGB2	4.651 (4.455 - 4.847)	4.615 (4.398 - 4.769)	4.753 (4.560 - 4.948)	<.001
JAM-A*	6.788 (6.339 - 7.242)	6.784 (6.382 - 7.188)	6.802 (6.277 - 7.373)	.45
KLK6	3.605 (3.354 - 3.886)	3.479 (3.285 - 3.686)	3.967 (3.709 - 4.207)	<.001
LDLR	3.893 (3.581 - 4.229)	3.872 (3.558 - 4.192)	3.969 (3.611 - 4.274)	.21
LTBR*	3.185 (2.967 - 3.524)	3.042 (2.877 - 3.194)	3.707 (3.512 - 4.075)	<.001
MB	6.300 (5.858 - 6.769)	6.178 (5.738 - 6.470)	6.788 (6.352 - 7.339)	<.001
MCP-1	4.068 (3.914 - 4.297)	4.023 (3.808 - 4.167)	4.266 (4.121 - 4.440)	<.001

MEPE	3.802 (3.492 - 4.131)	3.642 (3.379 - 3.902)	4.249 (3.880 - 4.492)	<.001
MMP-2	2.416 (2.168 - 2.653)	2.360 (2.119 - 2.575)	2.581 (2.276 - 2.852)	<.001
MMP-3	5.904 (5.562 - 6.359)	5.807 (5.436 - 6.100)	6.365 (5.825 - 6.666)	<.001
MMP-9	4.281 (3.715 - 4.816)	4.207 (3.635 - 4.803)	4.364 (3.773 - 4.850)	.54
MPO	2.304 (2.094 - 2.532)	2.255 (2.042 - 2.457)	2.422 (2.178 - 2.677)	<.001
Notch3	4.437 (4.248 - 4.711)	4.354 (4.146 - 4.557)	4.726 (4.426 - 4.947)	<.001
NT-proBNP	4.023 (3.089 - 5.030)	3.654 (2.933 - 4.504)	4.838 (3.977 - 5.931)	<.001
OPG	3.134 (2.955 - 3.407)	3.050 (2.881 - 3.212)	3.418 (3.203 - 3.561)	<.001
OPN	7.585 (7.244 - 7.941)	7.387 (7.085 - 7.663)	8.086 (7.742 - 8.594)	<.001
PAI*	5.868 (5.347 - 6.235)	5.862 (5.390 - 6.236)	5.917 (5.281 - 6.224)	.94
PCSK9	2.270 (2.078 - 2.466)	2.241 (2.050 - 2.421)	2.310 (2.104 - 2.526)	.052
PDGF subunit A	2.968 (2.624 - 3.414)	2.990 (2.704 - 3.383)	2.912 (2.453 - 3.466)	.52
PECAM-1*	5.011 (4.656 - 5.403)	4.996 (4.680 - 5.367)	5.057 (4.614 - 5.476)	.54
PGLYRP1	7.289 (7.008 - 7.678)	7.164 (6.906 - 7.457)	7.686 (7.287 - 8.158)	<.001
PI3	2.436 (2.039 - 2.970)	2.226 (1.893 - 2.609)	2.966 (2.567 - 3.574)	<.001
PLC	7.454 (7.179 - 7.820)	7.309 (7.065 - 7.474)	7.944 (7.815 - 8.119)	<.001
PON3	4.563 (4.188 - 5.030)	4.481 (4.063 - 4.862)	4.827 (4.414 - 5.135)	.004
PRTN3	3.893 (3.566 - 4.233)	3.797 (3.434 - 4.106)	4.140 (3.820 - 4.572)	<.001
PSP-D	2.552 (2.165 - 3.068)	2.506 (2.098 - 2.985)	2.764 (2.370 - 3.373)	.007
RARRES2	10.964 (10.721 - 11.159)	10.840 (10.678 - 11.044)	11.162 (11.014 - 11.352)	<.001
RETN	6.393 (6.106 - 6.819)	6.248 (6.018 - 6.467)	6.871 (6.619 - 7.373)	<.001
SCGB3A2	2.176 (1.699 - 2.735)	2.043 (1.563 - 2.637)	2.376 (2.012 - 2.916)	.001
SELE	9.606 (9.200 - 10.024)	9.581 (9.162 - 9.937)	9.736 (9.328 - 10.136)	.041
SELP*	10.124 (9.667 - 10.547)	10.108 (9.665 - 10.464)	10.144 (9.759 - 10.645)	.35
SHPS-1	3.205 (2.876 - 3.428)	3.087 (2.778 - 3.266)	3.472 (3.253 - 3.729)	<.001
ST2	6.106 (5.848 - 6.447)	6.044 (5.715 - 6.289)	6.253 (5.994 - 6.634)	<.001
TFF3	5.956 (5.690 - 6.408)	5.790 (5.530 - 6.014)	6.619 (6.284 - 6.954)	<.001
TFPI	8.553 (8.391 - 8.756)	8.502 (8.255 - 8.634)	8.730 (8.551 - 8.928)	<.001
TIMP4	4.298 (3.967 - 4.626)	4.210 (3.854 - 4.469)	4.556 (4.251 - 4.789)	<.001
TLT-2	4.546 (4.349 - 4.798)	4.495 (4.246 - 4.667)	4.817 (4.584 - 5.163)	<.001
TNF-R1*	5.936 (5.661 - 6.373)	5.753 (5.527 - 5.947)	6.591 (6.243 - 6.960)	<.001
TNF-R2*	4.636 (4.407 - 5.035)	4.513 (4.294 - 4.641)	5.290 (4.933 - 5.761)	<.001
TNFRSF10C	3.908 (3.646 - 4.234)	3.809 (3.553 - 4.097)	4.105 (3.833 - 4.537)	<.001
TNFRSF14	4.500 (4.288 - 4.829)	4.352 (4.173 - 4.518)	4.949 (4.753 - 5.293)	<.001
TNFSF13B	6.412 (6.238 - 6.654)	6.361 (6.177 - 6.531)	6.603 (6.382 - 6.859)	<.001
t-PA	4.738 (4.324 - 5.230)	4.651 (4.264 - 5.123)	4.870 (4.399 - 5.385)	.063
TR	4.477 (4.087 - 4.929)	4.349 (4.011 - 4.686)	4.806 (4.462 - 5.359)	<.001
TR-AP	4.049 (3.819 - 4.312)	3.978 (3.784 - 4.222)	4.212 (3.986 - 4.439)	<.001
uPA	4.691 (4.529 - 4.903)	4.643 (4.488 - 4.810)	4.890 (4.658 - 5.017)	<.001
U-PAR	4.732 (4.521 - 5.015)	4.582 (4.389 - 4.753)	5.195 (4.955 - 5.445)	<.001
vWF	5.856 (5.336 - 6.391)	5.787 (5.229 - 6.308)	6.230 (5.560 - 6.564)	.001

Biomarkers are expressed in Normalized Protein eXpression (NPX), which are relative units that result from the polymerase chain reaction and which are on the log2 scale. A high NPX value equals a high biomarker concentration and, because of the log2 scale, one unit difference in NPX represents a doubling of the protein concentration. All biomarkers are expressed as median (25th- 75th percentile).

* For highly correlated biomarker pairs (Pearson's correlation coefficient >0.8), the biomarker with the highest mean correlation was excluded. Accordingly,11 biomarkers were excluded from the cluster analysis in the EVAR group: CASP-3, CPB1, EPHB4, GP6, JAM-A, LTBR, PAI, PECAM-1, SELP, TNF-R1, TNF-R2.

Table S4. Clinical characteristics of watchful waiting patients according to cluster membership

	**Cluster 1** n=62	**Cluster 2** n=48	*p*-value
Age	70.5 ± 6.4	73.4 ± 7.6	.034
Male	57 (91.9)	41 (85.4)	.28
BMI	27.7 ± 3.3	27.6 ± 4.5	.81
Medical history			
Coronary heart disease*	23 (37.1)	18 (37.5)	.97
Heart Failure	3 (4.8)	5 (10.4)	.29
Hypertension	40 (64.5)	38 (79.2)	.093
Cerebrovascular disease	9 (14.5)	7 (14.6)	.99
Diabetes mellitus	15 (24.2)	12 (25.0)	.92
Peripheral arterial occlusive disease	12 (19.4)	12 (25.0)	.48
Chronic obstructive pulmonary disease	8 (12.9)	12 (25.0)	.10
Smoking			1.00
Never	2 (3.2)	1 (2.1)	
Current	20 (32.3)	15 (31.2)	
Former	40 (64.5)	32 (66.7)	
Medication use			
Antiplatelet	45 (72.6)	31 (64.6)	.37
Non-vitamin K oral anticoagulant (NOAC)	4 (6.5)	2 (4.2)	.69
Coumarin	3 (4.8)	9 (18.8)	.020
Beta-blocker	31 (50.0)	19 (39.6)	.28
Angiotensin converting enzyme (ACE) inhibitor	17 (27.4)	14 (29.2)	.84
Angiotensin II receptor antagonist	17 (27.4)	16 (33.3)	.50
Thiazide diuretic	7 (11.3)	14 (29.2)	.018
Lipid-lowering drug	47 (75.8)	38 (79.2)	.68
Aneurysm related information			
Anatomical classification AAA - infrarenal	59 (95.2)	45 (93.8)	.75
Maximal diameter AAA (mm)	50.0 (47.0 - 53.0)	49.0 (46.0 - 52.3)	.55
AAA volume (mL)	107.2 (94.2 - 128.7)	99.8 (90.2 - 130.0)	.52
Concurrent iliac artery aneurysm	12 (19.4)	6 (12.5)	.34

Continuous variables with a normal distribution are presented as mean ± standard deviation (SD), or median (25th - 75th percentile) in case of non-normality. Differences in baseline characteristics between patients in the two clusters were tested with Student t-tests or Mann Whitney U tests depending on their distributions. Categorical variables are presented as counts and percentages and differences between groups were tested using the chi-square test or Fisher's exact test, as appropriate.

*Coronary heart disease: history of myocardial infarction, and/or percutaneous coronary intervention and/or coronary artery bypass grafting.

AAA: abdominal aortic aneurysm.

Table S5. Clinical characteristics of EVAR patients according to cluster membership

	Cluster 1<br>n=136	Cluster 2<br>n=67	*p*-value
Age	73.1 ±7.4	76.7 ±7.6	.001
Male	128 (94.1)	59 (88.1)	.13
BMI	26.8 ±3.8	26.8 ±3.7	.99
Medical history			
Coronary heart disease*	44 (32.4)	25 (37.3)	.48
Heart Failure	6 (4.4)	7 (10.4)	.099
Hypertension	104 (76.5)	58 (86.6)	.092
Cerebrovascular disease	27 (19.9)	11 (16.4)	.56
Diabetes mellitus	22 (16.2)	13 (19.4)	.57
Peripheral arterial occlusive disease	18 (13.2)	10 (14.9)	.74
Chronic obstructive pulmonary disease	33 (24.3)	15 (22.4)	.77
Smoking			.57
Never	8 (5.9)	2 (3.0)	
Current	33 (24.3)	20 (29.9)	
Former	95 (69.9)	45 (67.2)	
Medication use			
Antiplatelet	108 (79.4)	50 (74.6)	.44
Non-vitamin K oral anticoagulant (NOAC)	8 (5.9)	1 (1.5)	.28
Coumarin	14 (10.3)	11 (16.4)	.21
Beta-blocker	68 (50.0)	48 (71.6)	.003
Angiotensin converting enzyme (ACE) inhibitor	36 (26.5)	24 (35.8)	.17
Angiotensin II receptor antagonist	38 (27.9)	20 (29.9)	.78
Thiazide diuretic	27 (19.9)	14 (20.9)	.86
Lipid-lowering drug	113 (83.1)	54 (80.6)	.66
Aneurysm related information			
Anatomical classification AAA - infrarenal	123 (90.4)	58 (86.6)	.40
Maximal diameter AAA (mm)	55.0 (45.8 - 63.7)	56.0 (45.0 - 67.0)	.35
Sac volume (mL)	142.1 (103.1 - 197.5)	157.7 (113.8 - 241.6)	.17
Concurrent iliac artery aneurysm	40 (29.4)	16 (23.9)	.41
Surgery related information			
Type of index surgery			.82
Primary EVAR	114 (83.8)	57 (85.1)	
Reintervention	22 (16.2)	10 (14.9)	
AAA ruptured at admission	9 (6.6)	5 (7.5)	.82
ASA classification			.58
ASA I or II	57 (41.9)	24 (35.8)	
ASA III, IV or IV	66 (48.5)	34 (5.7)	
Unknown	13 (9.6)	9 (13.4)	

Continuous variables with a normal distribution are presented as mean ± standard deviation (SD), or median (25th - 75th percentile) in case of non-normality. Differences in baseline characteristics between patients in the two clusters were tested with Student t-tests or Mann Whitney U tests depending on their distributions. Categorical variables are presented as counts and percentages and differences between groups were tested using the chi-square test or Fisher's exact test, as appropriate.

*Coronary heart disease: history of myocardial infarction, and/or percutaneous coronary intervention and/or coronary artery bypass grafting.

AAA: abdominal aortic aneurysm, ASA classification: Physical Status Classification System by the American Society of Anesthesiologists, EVAR: Endovascular Aneurysm Repair.

Table S6. Sensitivity analysis on the association of biomarkers with aneurysm sac volume in EVAR patients - adjusted for confounders

	A			B			C			D		
	β	95% CI	Adj. *p*-value	β	95% CI	Adj. *p*-value	β	95% CI	Adj. *p*-value	β	95% CI	*p*-value
IGFBP2	0.168	(0.078, 0.258)	.001	0.136	(0.047, 0.226)	.012	0.162	(0.068, 0.255)	.003	0.050	(-0.062, 0.163)	.376
LDLR	-0.151	(-0.237, -0.064)	.001	-0.116	(-0.203, -0.029)	.013	-0.117	(-0.205, -0.028)	.011	-0.149	(-0.242, -0.057)	.002
TFPI	0.149	(0.062, 0.237)	.001	0.122	(0.036, 0.208)	.012	0.136	(0.041, 0.232)	.011	0.146	(0.034, 0.257)	.011
TIMP4	0.140	(0.045, 0.235)	.004	0.110	(0.017, 0.203)	.021	0.126	(0.030, 0.222)	.011	0.057	(-0.045, 0.159)	.270

The statistical analyses were repeated while excluding patients who initially underwent EVAR for ruptured AAA and patients with a re-intervention between the EVAR and current measurements.
Linear regression analysis was applied to examine associations of individual biomarkers with sac volume.
A) Biomarkers with an adjusted p-value <.05 in univariable linear regression were adjusted for age, sex and body surface area.
B) Additionally, time between EVAR surgery and study visit was also added to the model.
C) Next, additional pre-defined variables were added: history of coronary heart disease, hypertension, diabetes mellitus, smoking status, peripheral arterial occlusive disease, antiplatelet therapy, lipid lowering drug therapy and familial abdominal aortic aneurysm.
D) Lastly, the four biomarkers of interest, together with the covariates from model C, were used to construct an adjusted multimarker model.

Results are presented as the mean effect with 95% confidence interval (CI) of a 1 standard deviation (SD) difference of the biomarker on sac volume (expressed as $\log_e$ mL) .
We corrected for multiple testing concluding significance with a Benjamini-Hochberg adjusted p-value <.05.

Abbreviations used: adj. *p*-value: adjusted *p*-value, β: regression coefficient, CI: confidence interval.

Table S7. Analysis on the association of biomarkers with aneurysm sac growth in EVAR patients - adjusted for confounders

	A			B			C		
	β	95% CI	*p*-value	β	95% CI	*p*-value	β	95% CI	*p*-value
LDLR	-3.235	(-5.150, -1.319)	.001	-2.346	(-4.268, -0.423)	.017	-2.527	(-4.486, -0.569)	.012
IGFBP2	1.152	(-0.93, 3.234)	.277	0.111	(-1.952, 2.173)	.916	0.404	(-1.731, 2.540)	.709
TFPI	1.562	(-0.435, 3.558)	.125	0.915	(-1.030, 2.861)	.355	1.521	(-0.598, 3.639)	.158
TIMP4	1.376	(-0.645, 3.397)	.181	0.723	(-1.245, 2.691)	.469	0.721	(-1.313, 2.755)	.485

The statistical analyses were repeated using sac growth defined as absolute difference between current diameter and preoperative diameter.
Linear regression analysis was applied to examine associations of individual biomarkers with sac growth.
A) Biomarkers with an adjusted p-value <.05 in univariable linear regression were adjusted for age, sex and body surface area. Additionally, the biomarkers of interest from the sac volume model in the EVAR group are also shown, to allow comparison between the sac volume and sac growth results.
B) Additionally, time between EVAR surgery and study visit was also added to the model.
C) Next, additional pre-defined variables were added: history of coronary heart disease, hypertension, diabetes mellitus, smoking status, peripheral arterial occlusive disease, antiplatelet therapy, lipid lowering drug therapy and familial abdominal aortic aneurysm.

Results are presented as the mean effect with 95% confidence interval (CI) of a 1 standard deviation (SD) difference of the biomarker on sac growth (expressed as mm).
As only one marker was selected by univariate linear regression and the other markers are merely illustratively portrayed, we did not correct the p-values for multiple testing.

Abbreviations used: β: regression coefficient, CI: confidence interval.
