# Supplementary material for: Circulating biomarkers of cardiovascular disease are related to aneurysm volume in abdominal aortic aneurysm
Source: Vasc Med. 2023 Jul 3;28(5):433–42. doi: 10.1177/1358863X231181159 (PMC10559648; doi:10.1177/1358863X231181159)
Supplement: sj-pdf-2-vmj-10.1177_1358863X231181159 – Supplemental material for Circulating biomarkers of cardiovascular disease are related to aneurysm volume in abdominal aortic aneurysm [file sj-pdf-2-vmj-10.1177_1358863X231181159.pdf]

SUPPLEMENTAL MATERIAL

Circulating biomarkers of cardiovascular disease are related to aneurysm volume in abdominal aortic aneurysm patients

Supplemental methods

Study design

The BIOMArCS-AAA study consists of 2 prospective arms and 1 cross-sectional arm. The 2 prospective arms include: I) patients with a clinical diagnosis of an AAA of 40mm or more who are under watchful waiting (periodic clinical observation, no need for surgical aneurysm in the nearby future – 'watchful waiting patients') and II) patients who are scheduled to undergo EVAR for AAA in the nearby future ('longitudinal EVAR patients'). The cross-sectional arm consists of patients who have previously undergone EVAR for AAA in the past years ('cross-sectional EVAR patients'). All patients had to be of 18 years of age or older and in the capacity to understand and give informed consent. Exclusion criteria were patients with a thoracic aortic aneurysm, a saccular, traumatic, anastomotic or infectious abdominal aortic aneurysm or patients with an isolated iliac artery aneurysm. Patients with a life expectancy less than 1 year were excluded, as well as dialysis dependent patients or patients with end stage renal disease (eGFR <15 ml/min). Women of childbearing age were not included. Patients with a linguistic barrier or who were unlikely to appear at all scheduled follow-up visits were also excluded (supplementary Figure S1).

The prospective arms have a 24-month follow-up period with clinical data collection and blood sampling at baseline, at 1 month after EVAR and at 6, 12, 18 and 24 months for all patients. CT is conducted at baseline and 12 and 24 months, and additionally at 1 month in the EVAR patients. For the cross-sectional arm, clinical data collection, blood sampling, ultrasound and CT is performed once after EVAR. This is depicted in Supplementary Figure S1. We used the baseline visit samples of the watchful waiting patients; as well as the samples at first study visit available after surgery of the EVAR patients (the latter consisting of both patients from longitudinal as well cross-sectional arm). From the 340 available patients, 22 were excluded due to incomplete imaging and/or incomplete or absent blood sample (Supplementary Figure S2a), leaving 316 patients for laboratory analysis.

Sample size

For the cross-sectional arm, a sample size of 200 patients would be required in order to demonstrate an effect of 0.36 on standardized volume of the aneurysm sac (difference divided by standard deviation) for any biomarker, comparing the samples with a biomarker value above and below the

median. For both longitudinal arms with 5 repeated biomarker measurements, sac growth (volume increase of at least 5%) was expected in 10% of the patients. Assuming a linear association and a continuous autoregressive correlation matrix, 500 simulations were ran for the sample size calculation, using the measurement error (sigma) and input parameter (rho) of the repeated LDL measurements from a previous patient cohort. A sample size of 120 patients in each longitudinal arm would be sufficient to demonstrate a difference of 0.02 mmol/L between cases of sac growth and non-cases in monthly change in biomarker level, assuming a power of 80% and alpha of 0.05. These longitudinal sample sizes would also allow to demonstrate a difference of 0.51 in standardized sac volume for any biomarker (comparing samples above and below the median biomarker value) at any moment in time.

Blood sample processing and quality control

One µL of each of the 316 EDTA plasma samples were diluted 1:100 and used to assess the abundance of circulating proteins using proximity extension assay (PEA) technology on six plates. PEA is based on dual-recognition immunoassay. Antibodies are labelled with unique DNA oligonucleotides, which hybridize when brought in proximity by binding to their specific protein. These hybridized oligonucleotides serve as the unique template during polymerase chain reaction amplification. The specific DNA amplicon is quantified by qPCR (Fluidigm® BioMark system). As the concentration of a specific amplicon is proportional to the concentration of hybridized oligonucleotides, which in turn is determined by the concentration of protein, PEA is able to measure relative protein abundance.[1, 2]

Two external controls are added to each plate, to achieve consistency across assays. To achieve consistent normalization across plates, a first external control consists of 92 antibodies with one of the pairs of unique DNA oligonucleotides in fixed proximity. The second, negative control, consists of antigen-free buffer and is used to set background levels.

Additionally, the PEA process is monitored by four internal controls, added to each sample. These include two incubation control, which are two non-human antigens to monitor the incubation protocol and thus all downward steps; one extension control, which is an antibody with both its DNA-tags always in proximity to monitor the hybridization and is used for normalization across samples;

and one detection control, which is an amplicon to monitor the amplification and quantification steps.[1, 2]

Sample plates were deemed of sufficient quality if the internal controls had a standard deviation of less than 0.2 normalized protein expression (NPX, log_2 relative protein abundance). Individual sample quality was sufficient when samples deviated less than 0.3 NPX from the median of the internal controls in that sample.

Statistical analysis – additional information on clustering and heatmap

We performed k-means clustering to examine presence of subgroups (within the watchful waiting and EVAR group separately) based on biomarker profile.[3] First, Pearson's correlation coefficients were calculated between the 91 biomarkers. For highly correlated biomarker pairs (coefficient >0.8), the biomarker with the highest correlation coefficient was excluded. Accordingly,13 biomarkers were excluded from the cluster analysis in the watchful waiting group and 11 in the EVAR group. The optimal number of clusters was defined using the NbClust package (distance set as euclidean, method set as kmeans) after which k-means clustering was performed to divide patients into clusters. Clusterwise stability was assessed with the function clusterboot in R (B set as 1000, clustermethod set as kmeansCBI) and clusterwise stability as assessed by Jaccard similarity values was high (watchful waiting group cluster 1=0.96 and cluster 2=0.95; EVAR group cluster 1=0.97 and cluster 2=0.94). We examined the distribution of clinical characteristics and aneurysm related measurements according to the clusters. Differences between clusters were tested with Student t-tests or Mann Whitney U tests depending on variable distributions.

We used heatmaps to visualize the biomarker results for each cluster (defined with k-means clustering, as described above). These heatmaps depict biomarker values for all individual patients that are transformed to a color scale and are clustered in both rows and columns. To acquire the heatmaps, dissimilarity matrices, in which the distance or (dis)similarity between each pair of observations is computed, were obtained based on Euclidean distance. With the linkage function (complete method), patients were grouped according to a hierarchical tree based on the (dis)similarity information resulting in reordering in such a way that highly similar observations were neighbored.

REFERENCES

1. Olink Proteomics. PEA – a high-multiplex immunoassay technology with qPCR or NGS readout 2020 [Available from: https://www.olink.com/content/uploads/2021/09/olink-white-paper-pea-a-high-multiplex-immunoassay-technology-with-qpcr-or-ngs-readout-v1.0.pdf, Accessed January 15 2023.

2. Olink Proteomics. Olink Target 96 User Manual 2022 [Available from: https://www.olink.com/content/uploads/2022/04/olink-target-96-user-manual.pdf, Accessed January 15 2023.

3. Kassambara A. Practical Guide to Cluster Analysis in R: Unsupervised Machine Learning (Multivariate Analysis). 1st edition ed: STHDA; 2017.
