# Supplementary material for: Circulating biomarkers of cardiovascular disease are related to aneurysm volume in abdominal aortic aneurysm
Source: Vasc Med. 2023 Jul 3;28(5):433–42. doi: 10.1177/1358863X231181159 (PMC10559648; doi:10.1177/1358863X231181159)

Supplemental Figure S1. Flowchart

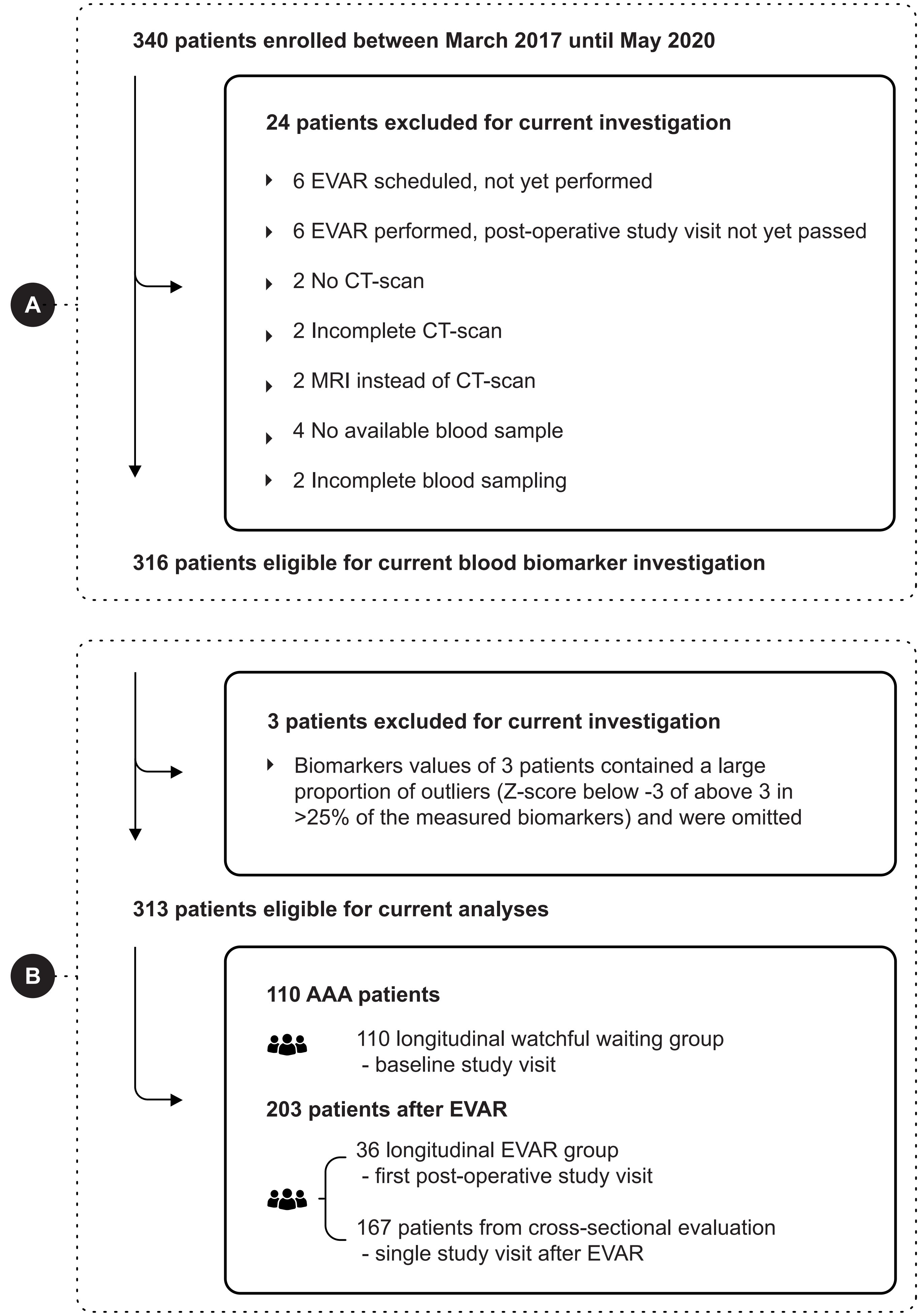

Supplemental Figure S2. Difference in biomarker levels between watchful waiting and post-EVAR patients

Post-EVAR patients vs. watchful waiting patients

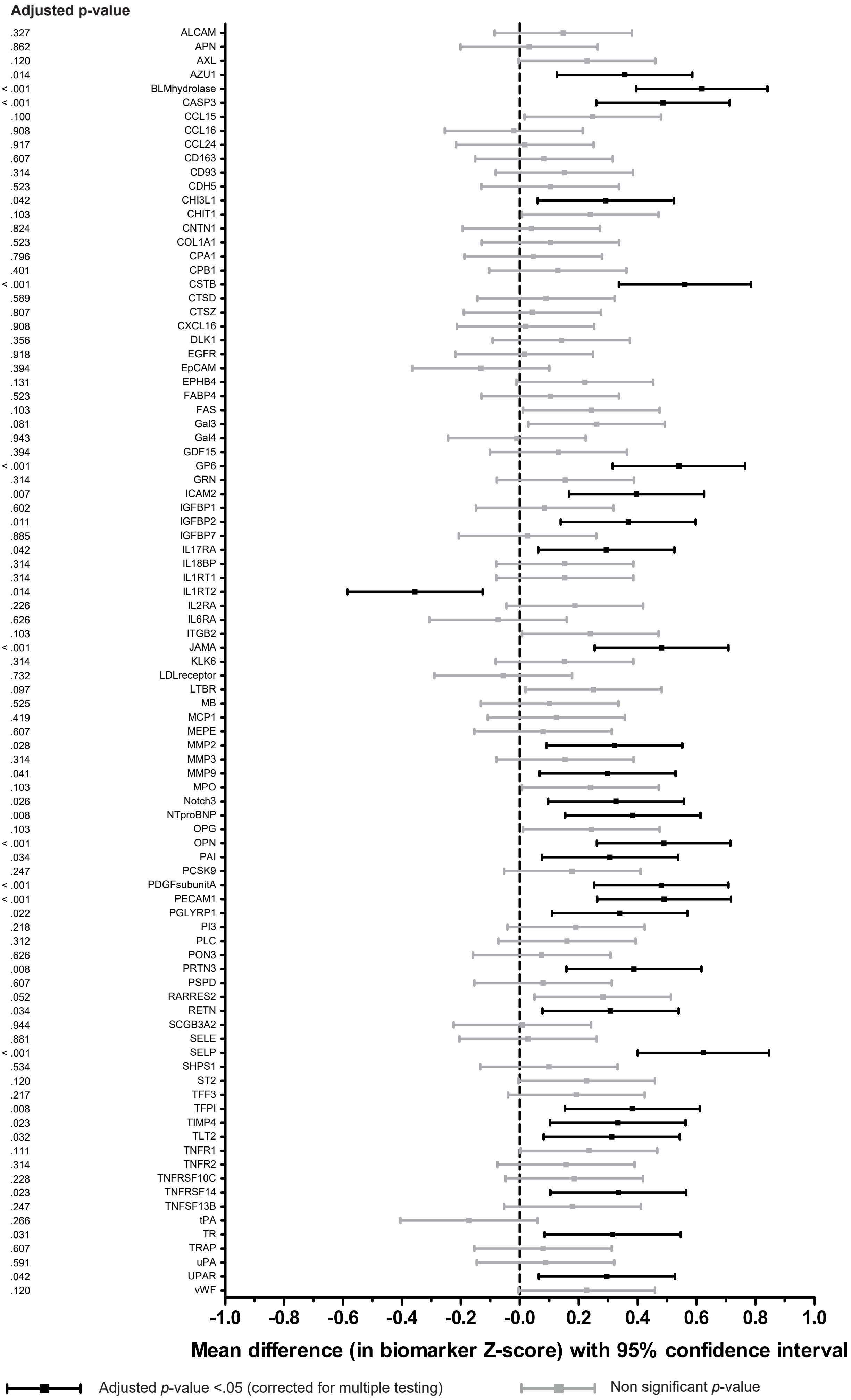

Supplementary figure S3. Distribution of Time Interval between EVAR and Current Measurements

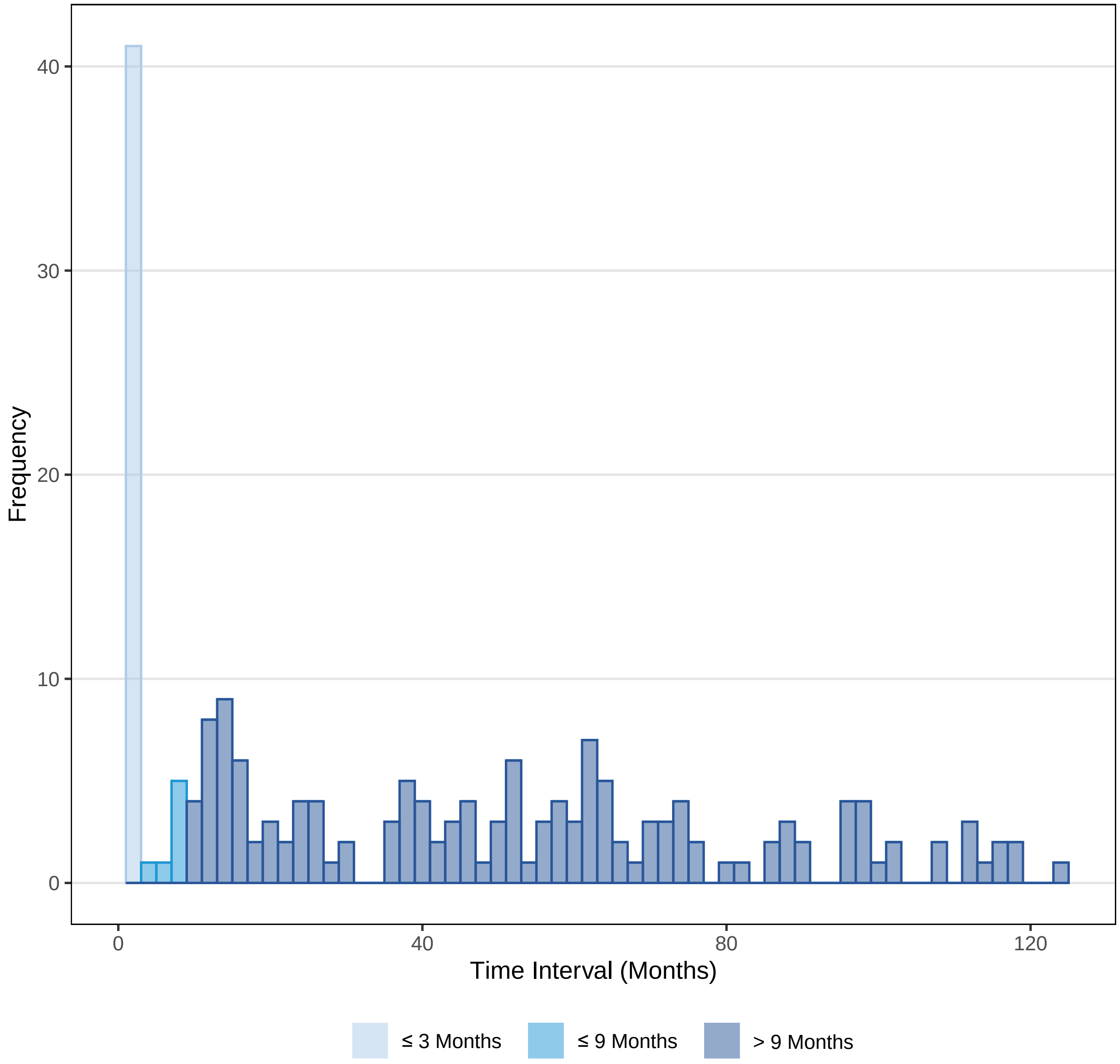

Supplementary figure S4. Difference in biomarker levels according to time after EVAR procedure

Blood sampling <90 days after EVAR vs. blood sampling >90 days after EVAR

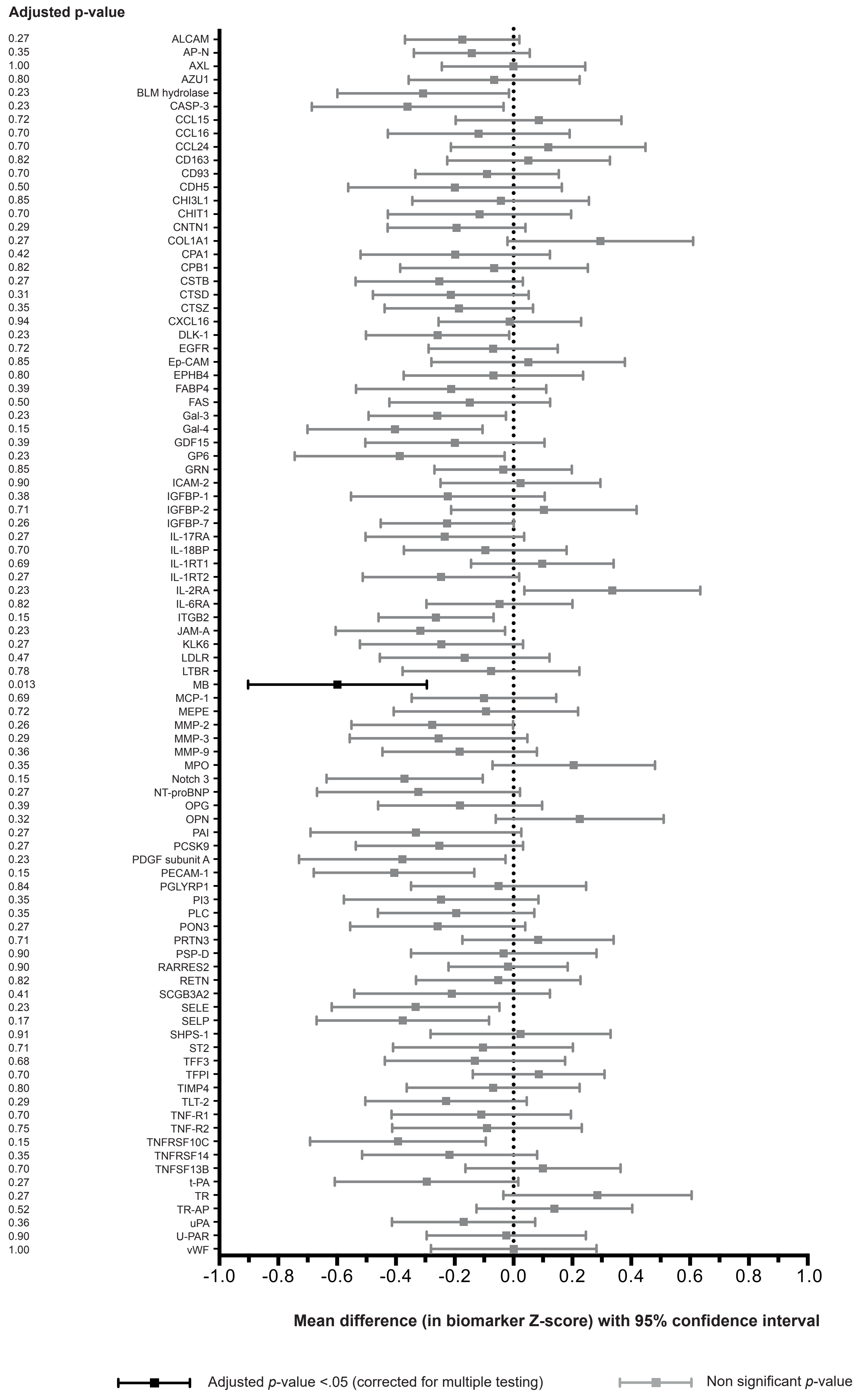

Supplemental Figure S5(A). Clustering in watchful waiting patients based on biomarker values

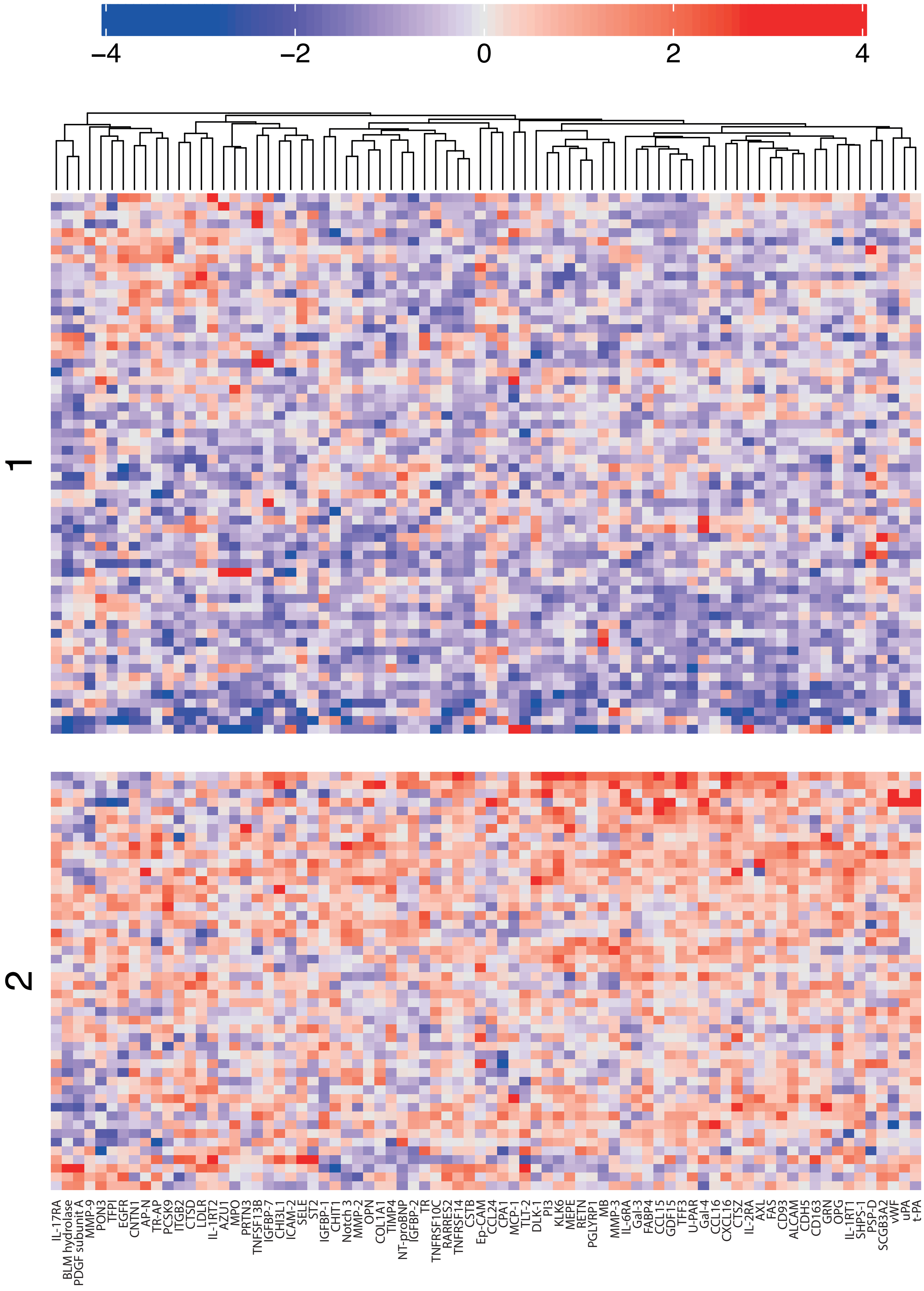

Supplemental Figure S5(B). Clustering in patients after EVAR, based on biomarker values

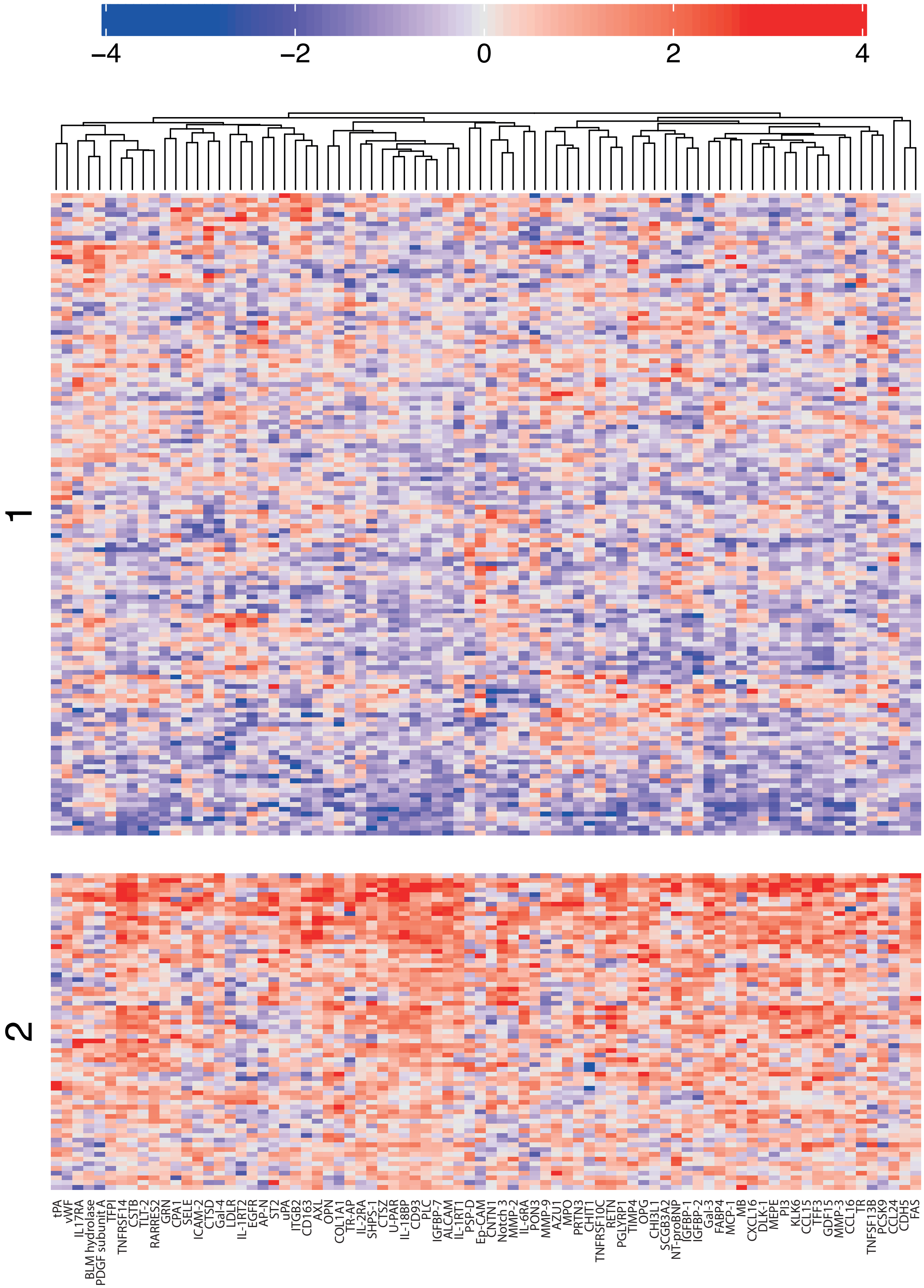

Supplementary Figure S6. Association of biomarkers with sac growth in patients after EVAR

Biomarker	β	95% CI
ALCAM	-0.799	(-2.68 1.082)
APN	0.832	(-0.954 2.618)
AXL	-0.058	(-1.93 1.814)
AZU1	0.271	(-1.685 2.227)
BLMhydrolase	0.537	(-1.533 2.607)
CASP3	0.146	(-1.868 2.159)
CCL15	-0.2	(-2.085 1.684)
CCL16	-0.579	(-2.381 1.224)
CCL24	-1.419	(-3.214 0.376)
CD163	-0.396	(-2.22 1.428)
CD93	-1.159	(-3.026 0.709)
CDH5	-0.061	(-1.694 1.571)
CHI3L1	1.076	(-0.892 3.044)
CHIT1	0.554	(-1.406 2.513)
CNTN1	-0.831	(-2.7 1.039)
COL1A1	0.88	(-0.924 2.683)
CPA1	-1.477	(-3.309 0.354)
CPB1	-1.805	(-3.644 0.035)
CSTB	-0.231	(-2.143 1.68)
CTSD	-0.238	(-2.158 1.683)
CTSZ	-1.072	(-3.023 0.88)
CXCL16	0.336	(-1.543 2.214)
DLK1	-1.607	(-3.668 0.454)
EGFR	-1.839	(-3.84 0.161)
EpCAM	-0.932	(-2.763 0.9)
EPHB4	-0.588	(-2.363 1.187)
FABP4	-0.854	(-2.662 0.954)
FAS	-0.609	(-2.341 1.123)
Gal3	-0.347	(-2.16 1.466)
Gal4	-0.328	(-2.208 1.552)
GDF15	1.005	(-0.842 2.851)
GP6	0.569	(-1.416 2.555)
GRN	-1.001	(-2.87 0.868)
ICAM2	1.235	(-0.689 3.159)
IGFBP1	0.68	(-1.205 2.565)
IGFBP2	1.686	(-0.137 3.508)
IGFBP7	-0.105	(-2.167 1.958)
IL17RA	1.267	(-0.635 3.17)
IL18BP	-0.994	(-2.842 0.855)
IL1RT1	1.01	(-0.857 2.876)
IL1RT2	-0.836	(-2.715 1.042)
IL2RA	0.15	(-1.692 1.993)
IL6RA	-0.961	(-2.857 0.934)
ITGB2	-1.09	(-3.024 0.844)
JAMA	0.425	(-1.574 2.424)
KLK6	-1.058	(-2.993 0.876)
LDLreceptor	-3.374	(-5.221 -1.528)
LTBR	-0.191	(-2.02 1.639)
MB	-2.151	(-3.984 -0.319)
MCP1	-1.221	(-3.243 0.801)
MEPE	-1.002	(-2.819 0.815)
MMP2	-0.318	(-2.134 1.498)
MMP3	-2.011	(-3.863 -0.158)
MMP9	-1.221	(-3.085 0.643)
MPO	1.223	(-0.782 3.227)
Notch3	0.081	(-1.828 1.989)
NTproBNP	1.167	(-0.627 2.962)
OPG	2.136	(0.274 3.997)
OPN	1.615	(-0.292 3.521)
PAI	-0.122	(-2.051 1.808)
PCSK9	-0.407	(-2.356 1.543)
PDGFsubunitA	0.556	(-1.471 2.584)
PECAM1	0.186	(-1.858 2.23)
PGLYRP1	-0.454	(-2.214 1.305)
PI3	-0.425	(-2.276 1.426)
PLC	-0.736	(-2.605 1.133)
PON3	0.124	(-1.762 2.01)
PRTN3	0.175	(-1.772 2.122)
PSPD	-1.521	(-3.446 0.404)
RARRES2	-0.163	(-2.029 1.703)
RETN	-0.916	(-2.775 0.943)
SCGB3A2	0.378	(-1.492 2.248)
SELE	-1.484	(-3.359 0.391)
SELP	-0.153	(-2.238 1.932)
SHPS1	0.102	(-1.704 1.908)
ST2	0.81	(-1.011 2.631)
TFF3	0.127	(-1.683 1.936)
TFPI	1.765	(-0.207 3.738)
TIMP4	1.649	(-0.227 3.524)
TLT2	-0.216	(-2.295 1.863)
TNFR1	-0.329	(-2.174 1.516)
TNFR2	-0.28	(-2.107 1.547)
TNFRSF10C	-1.419	(-3.233 0.394)
TNFRSF14	-0.459	(-2.3 1.382)
TNFSF13B	1.93	(-0.023 3.883)
tPA	-2.086	(-3.945 -0.227)
TR	1.811	(-0.034 3.655)
TRAP	0.203	(-1.701 2.107)
uPA	0.972	(-0.873 2.817)
UPAR	0.47	(-1.348 2.288)
vWF	-1.157	(-3.156 0.842)

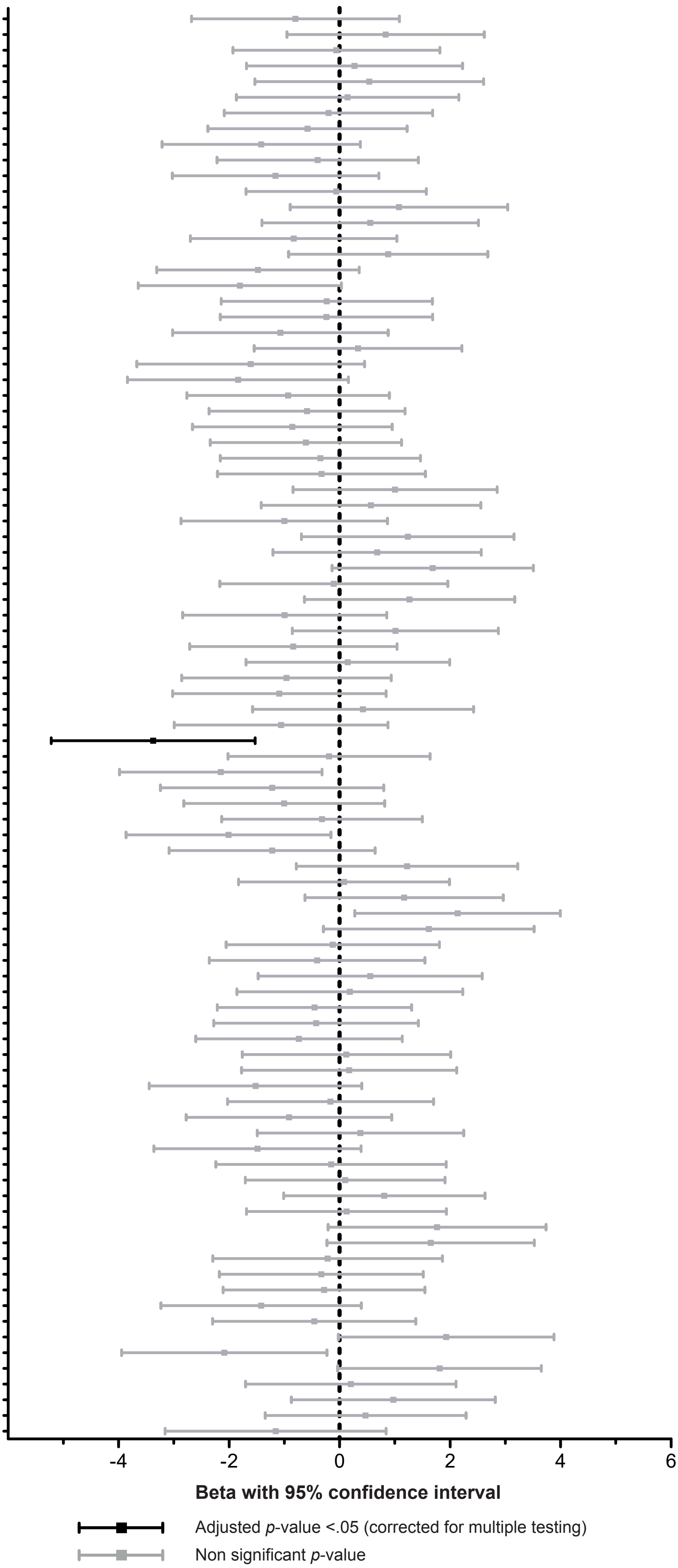

Supplement: sj-pdf-3-vmj-10.1177_1358863X231181159 – Supplemental material for Circulating biomarkers of cardiovascular disease are related to aneurysm volume in abdominal aortic aneurysm [file sj-pdf-3-vmj-10.1177_1358863X231181159.pdf]
